# Psychometric validation of the continuum beliefs of mental illness scale (CB-MIS) and its associations with stigma

**DOI:** 10.1186/s12888-024-06467-8

**Published:** 2025-02-04

**Authors:** Lina-Jolien Peter, Thomas McLaren, Samuel Tomczyk, Holger Muehlan, Silke Schmidt, Georg Schomerus

**Affiliations:** 1 Department of Psychiatry and Psychotherapy, Medical Faculty, University Leipzig, Semmelweisstr. 10, 04103 Leipzig, Germany; 2 Department of Psychiatry and Psychotherapy, University of Leipzig Medical Center, Leipzig, Germany; 3 Department of Health and Prevention, Institute of Psychology, University of Greifswald, Greifswald, Germany; 4 Department of Medicine, Health & Medical University Erfurt, Erfurt, Germany

**Keywords:** Continuum beliefs; Mental Health Continuum, Scale Development, Mental Illness Stigma, Discrimination, Stereotypes, Anti-stigma

## Abstract

**Background:**

Continuum beliefs of mental health and illness are robustly negatively correlated with mental illness stigma. However, there is a lack of multidimensional and validated measures, not entirely relying on vignettes.

**Methods:**

To develop such a scale, a pool of 37 items adapted from other studies on continuum beliefs, was initially examined in expert discussions and a convenience sample (*N* = 227, *M*_age_=32 years, 80.6% female). Items were selected based on theoretical tenability and assigned to pre-defined facets of “*State*”, “*Person*”, and nosological “*Concept*” in relation to mental illness. In a second sample (*N* = 1375; *M*_age_=42 years; 65.2% female), the *Continuum Beliefs of Mental Illness Scale (CB-MIS)* was psychometrically tested (i.e. factorial, convergent, discriminant validity).

**Results:**

The scale comprises three subscales with three items, and one optional vignette-based item each, rated on a 5-point Likert scale. It showed very good factorial and discriminant validity, associations with stigma were moderately negative. The scale exhibited good test-retest reliability over a period of six months.

**Conclusions:**

A validated, practical, multifaceted measure is offered to evaluate beliefs regarding the continuum of mental health and illness. Future studies should conduct subgroup-specific investigations regarding sociodemographic and illness variables, and could apply this measure to anti-stigma interventions.

**Trial registration:**

German Clinical Trials Register: DRKS00023557. Registered 11/12/2020. World Health Organization, Universal Trial Number: U1111–1264–9954. Registered 16/02/2021.

**Supplementary Information:**

The online version contains supplementary material available at 10.1186/s12888-024-06467-8.

## Background

In contemporary discussions surrounding mental health conditions, due consideration should be given to the pervasive presence of and ongoing effort against stigmatizing attitudes [1]. Stigma encompasses prejudices, stereotypes, and discrimination directed towards groups of people, with the purpose of keeping „them“ down, out, or away [2]. The conceptualization of mental health and illness significantly influences the perceptions of individuals with mental illness, shaping the attitudes held by both the public [3] and affected individuals themselves [4]. Among individuals with depressive symptoms, 79% report experiences of discrimination throughout their lives [5]. Internalized stigma and discriminatory experiences detrimentally impact the well-being and recovery of those affected [6, 7].

The categorization of individuals due to various characteristics like health status, is inherently linked to the process of stigmatization [8].There are endeavours in different fields to overcome stigma as a source of societal inequity [9, 10]. Therefore, to enable a more comprehensive assessment of mental health conditions, clinical diagnostics are increasingly incorporating dimensional approaches [11–14].

Similarly, in the realm of public mental health, beyond the scope of diagnosis, continuum beliefs have been discussed as a dimensional nosological concept [15], that is, perceiving symptoms on a spectrum of lived experiences without social categorization due to mental health problems. In this sense, anyone can encounter phenomena of mental ill-health, and manifold opportunities for improvement are accessible, allowing movement along the continuum without falling into labelled categories. The concept of continuum assumes a more functional view of health, in line with the International Classification of Functioning, Disability, and Health (ICF). It contrasts categorical thinking in the area of mental health, such as essentialist beliefs [16]. Higher agreement with continuum beliefs is connected to lower stigma depending on categorization processes, both theoretically [8] as well as empirically [see 17, 18]. For the general population, continuum beliefs were overall associated with reduced social distance and increased pro-social behaviours [17]. The assessment of continuum beliefs of individuals with lived experience may provide new promising avenues to change stigma, and lately has also been examined regarding health-related and help-seeking variables [19].

In recent years, there has been a growing number of studies examining continuum beliefs in relation to stigma across different mental illnesses, contributing to intervention strategies [17]. Nevertheless, it is crucial to highlight a notable research gap in the assessment of continuum beliefs [17, 20]. The findings to date are largely based on single item assessments [18, 20]. Schomerus and colleagues, 2013 [15] established the item „Basically we are all sometimes like this person. It’s just a question how pronounced this state is.“, with agreement rated on a 5-point Likert scale [15]. The item usually followed a vignette briefly describing symptoms of a mental illness and sometimes containing personalization like name, gender, work place, etc., and a manipulation text/video on continuum beliefs [17]. This economical assessment enabled the inclusion of the continuum concept into a range of studies, and it has thus substantially expanded this research area [17].

However, the reliance on vignettes is seen as limitation due to the results depending highly on the chosen manipulation material [18]. Moreover, the existing measures with more items are often one-dimensional [21–23], neglecting the multidimensional implications of continuum beliefs.

In fact, Tomczyk and colleagues, 2022 [20] have identified several underlying constructs within the items of the summarized questionnaires that have not been further explored to date. These constructs include the *normality of being in a state of having mental health problems* and symptoms, reflecting whether experiences are lying on a continuum, and the *normality of the individuals with mental health problems*, focussing on how they are perceived in relation to others. The third aspect represents the *nosological concept of mental health and illness*, spanning from categorical to continuous conceptualizations of mental illness This third aspect aligns more closely with current perspectives on the dimensional diagnosis of mental illness (e.g., DSM 5).

Beyond different conceptualizations of the poles of a continuum scale, the “directions” of the scale in relation to the respondents—thereby influencing the perceived distance from the topic—could potentially affect associations with stigma [17]. A key difference could lie in whether participants are willing to position themselves on the same continuum of health and illness, with individuals experiencing mental health issues also placed within this spectrum [17], speaking of a personal, ‘inside perspective’. The opposite refers to a general, ‘outside perspective’, where a conceptual spectrum of mental health and illness is detached from one’s own experience, or where an individual is mentally positioned in a distinct group [8]. Hence, there might be an “*us-them/others*”-continuum, distinguishing whether someone is perceived as “like us” or “like them/others” [8], linked to the enhancement of either perceived *similarities*, or fundamental *differences* [24] between the respondents and the persons with mental health problems. These two conceptual poles likewise characterize how someone positions themself in relation to a person with mental illness, which evokes different associations with stigma [24]. In principle, the understanding of the continuum counteracts aspects of essentialist beliefs [16] that have previously been studied, disconnecting mental health complaints from personal identity (informativeness, inherence), diagnostic labels and entities (historical invariance, discreteness, naturalness), and the uniformity of mental disorders (uniformity), and instead focusing on the ubiquity of the experience (necessary features), and the road to recovery (immutability).

Examining the questionnaires utilized to assess continuum beliefs thus far reveals their strength in featuring relatively concise, efficiently deployable, and well-readable items [e.g. 25]. They are mostly developed theory-based, resulting in robust content and construct validity [e.g. 23]. However, comprehensive psychometric investigations, such as reliability and factorial validity, present a current gap in research [20].

**Fig. 1:**
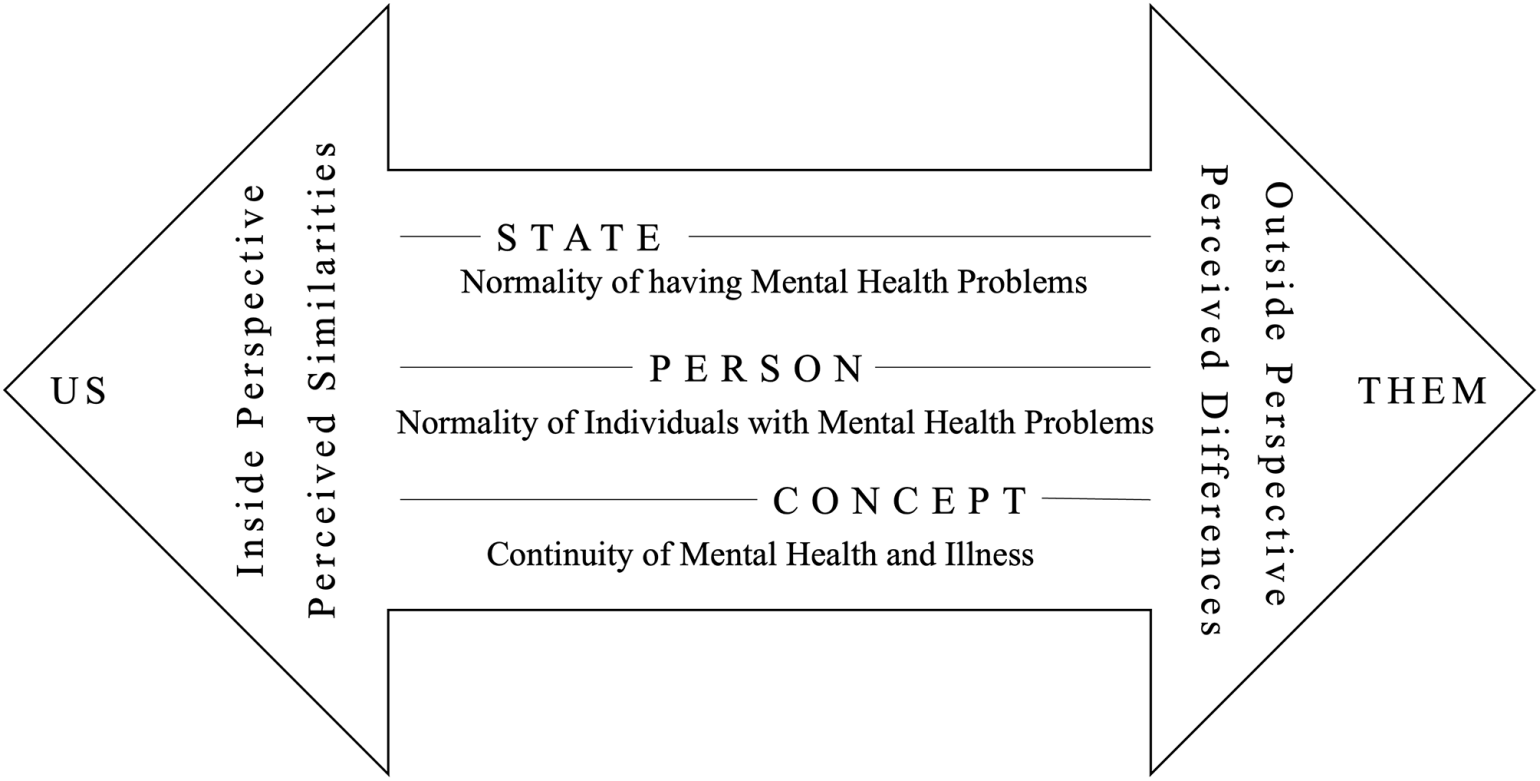
Continuum beliefs model conceptualization including a continuum of content facets

In sum, the study aims to establish a theoretically and empirically grounded, generic, multifaceted, validated, and practical measure of continuum beliefs of mental illness. The instrument should address several gaps. First, it should incorporate subconstructs related to the normality of being in a state of having mental health problems (hereafter called “*State*”), the normality of the individuals with mental health problems (hereafter called “*Person*”), and the nosological concept (hereafter called “*Concept*”). Moreover, the perspective expressed by the items (e.g., *inside* vs. *outside perspective*) and the implied distance (e.g. *similarities* vs. *differences* between respondents and people with mental illness) will be examined. Figure 1 summarizes the conceptualization of continuum beliefs for this study.

This questionnaire aims to capture two perspectives: on the one hand, the continuum beliefs that the general population holds about people with depression, and on the other hand, extending the literature, how individuals with symptoms of mental illness perceive general mental health and illness. Therefore, one sample will be drawn from the general population, while the other will specifically target individuals with depressive symptoms. We chose depressive symptoms because depression is a highly prevalent mental illness that has often been studied with regard to continuum beliefs in the general population [e.g. 26, 27]. For the development and validation of the scale, we follow the guidelines of Boateng and colleagues [28] to cover content validity, pre-tests, and psychometric validation. The measure will undergo thorough psychometric testing with emphasis placed on convergent validity examining associations with stigma. Regarding a sample exhibiting depressive symptoms, the associations with health-related variables will be examined to explore how continuum beliefs are connected to identification with mental illness, describing one’s symptoms and attitudes towards healthcare use. To transition from the current research landscape to a questionnaire suitable for both the general population and individuals with symptoms of mental illness, the study was conducted in two phases.

Phase 1: The first phase is based on existing literature, with rephrased items focused specifically on depression. The items target individuals from a general sample, similar to how continuum beliefs have been previously assessed. The structuring of the questionnaire and the reduction of items are carried out based on a content analysis.

Phase 2: The second phase introduces new aspects that expand upon existing research, rephrasing items to address general mental health and directing them towards a sample with depressive symptoms. It represents the psychometric main part of this manuscript and focuses on validating the questionnaire.

## Phase 1: Scale development

In this first part, the aim is to create a new multifaceted continuum beliefs scale from an item pool. At this stage, the items assess continuum beliefs of the general population about depression and affected individuals.

### Methods - phase 1

#### Item pool

In sum, 37 items on continuum beliefs (see Supplement S1.1) were collected from existing measures applied in previous studies [15, 22–24, 29–31], also including four unpublished items used in a prior study from our research group. Items were rated using Likert scales as described in the original publications, ranging from 1–4 to 1–7, representing a spectrum to state agreement with the statements (see table S1.1 for details). These items represent the majority of existing empirical assessment methods [20], covering a range of facets and features captured through the assessment of continuum beliefs in previous research. For quantitative data collection, the items were translated from English to German (TM, LJP), and adapted to focus on depressive symptoms where they did not already (TM, LJP), as shown in the table S1.1 in the supplement. Since some of the original items are based on vignettes, a labelled vignette (see supplement S1.1 for instructions and vignette) was presented before the assessment of all 37 items, providing a shared context for responding to them. The vignette, featuring ‘Alex,’ was designed to be gender-neutral and depicted depressive symptoms.

#### Content classification

To select items from the item pool to develop a new questionnaire, a content analysis was conducted. The 37 items were independently categorized by three authors (ST, HM, TM) followed by a discussion in the research team. Three content-similar categories of items were detected, reflecting the core aspects formulated by Tomczyk and colleagues, 2022 [20] pertaining to *State*, *Person*, and *Concept* (see Fig. 1). Items were grouped accordingly to three preliminary subscales.

The items were further clustered and rated regarding the inclusivity of the formulations and the potentially implied distance to the item subject during response. If the question ‘Must the respondent position themselves on the continuum of mental health and illness to answer this item?’ could be answered with ‘yes’ the label *‘Inside Perspective’* (e.g., “Most of us”**)** was assigned. If not, *‘Outside Perspective*’ (e.g., “other people”) was assigned, as shown in Fig. 1. Further, the perceived *Similarity*, e.g., „just like everyone else“ or *Differentness*, e.g., „fundamentally different from” was rated. This content analysis provided the foundation for selecting items for a new continuum beliefs scale, including the specified subscales. Our objective was to develop a scale that balances all content aspects and represents the various dimensions of a continuum belief model, as illustrated in Fig. 1.

From the entire pool of items, selections were made to represent the three facets, namely *State*, *Person*, and *Concept*, ensuring a meaningful combination of items without introducing redundancy. Items like the ones from Schomerus and colleagues from 2013 [15] and 2016 [24] were included in the item pool, as they belong to the most used items from literature, but some are very similar (e.g., item 1 and 2 in table S1.1). Subsequently, a subset of 16 items was examined, of which five items were allocated to the *State* facet, seven for the *Person* facet and four to the *Concept* facet. The classifications of formulations can be found in the supplement (table S1.1). To validate the item selections, facilitate further item reduction, and finalize the item choices for subsequent validation in Phase 2, item analyses were conducted during Phase 1. These psychometric analyses served as additional criteria alongside the content-based considerations, which served as the primary selection approach in Phase 1.

#### Survey Procedure and Sample

In Phase 1, psychometric analyses focused on a convenience sample (*N* = 227) that completed all items from the item pool. Participants were recruited via flyers and social media and completed an online survey lasting 30 to 45 min. They needed to be over 18 years old, with no additional inclusion criteria. Participants were offered the chance to win vouchers (6 valued between 10–30€) for a health food store as part of a study investigating reasons behind avoidance and strategies to promote mental health help-seeking. Data was collected in a pseudonymized manner, with the right to deletion ensured through a self-chosen password.

#### Measures

Table 1 contains the stigma and health-related variables. Sociodemographic data was furthermore gathered to provide information of the sample. Age, sex (female, male, diverse), income (from 0-500€ to ≥ 2500€, six levels), and education were assessed. Education is measured in years typically spent in school to achieve a common German school qualification. The completion of nine years of education leads to a basic graduation known as the “Hauptschulabschluss.” After ten years, students attain a middle graduation, referred to as the “Mittelschulabschluss.” Completion of secondary education, resulting in the “Abitur,” generally results from 12 to 13 years and signifies a higher qualification.

**Table 1:** Stigma and health-related variables measures

Variable	Scale	Scaling	Item example	α_sample 1_	α_sample 2_
*Stigmatizing attitudes*					
Agreement to stereotypes	German Version of the SSMIS-self-SF [32]	5 items, 1=“strongly disagree” to 5=“strongly agree	„I think … most people with mental illness are dangerous” “I think the public believes…”	0.83	0.80
Awareness of stereotypes	German Version of the SSMIS-public-SF [32]			0.92	0.84
Blame	Blame [24]	3 items, 1=“don’t agree at all” to 5=“agree completely”	“People with mental illness are to blame for their problems.”	0.78	0.84
Support for discriminative statements	Support for discriminative statements [33]	4 items, 1=“don’t agree at all” to 5=“agree completely”	“If persons with mental illness do not consent to medical treatment, they should receive compulsory treatment”	0.63	0.70
Shame	Shame of having a mental illness and seeking professional help [34]	2 items, 1=“not at all”, 2=“a little”, 3=“fairly”, 4=“fairly strong”, 5=“very strong”	“Would you feel shame, if you had mental problems?”	0.86	0.86
Desire of social distance from people with mental illness	Social distance scale, SDS [35]	7 items, 1=“very unlikely” to 5=“very likely”	“How willing would you be about renting a room in your home to a person with severe mental illness?”	-	0.89
Emotional reactions to a person with mental illness	ERMIS [36]	3 items each, 1=“don’t agree at all” to 5=“agree completely”			
	fear		“The person makes me feels afraid”	-	0.82
	anger		“I react annoyed”	-	0.80
	pro-social reaction		“I feel pity towards the person”	-	0.64
*Health-related variables*					
General health status	German version of the SF-36 health survey [37]	1 item, 1=“bad” to 5=“excellent”.	“How would you describe your overall health status?”		
Depression severity	German Version of the Patient Health Questionnaire, PHQ-9 [38]	9 items, 1 = “Not at all” – 4 = “nearly every day”, sum score from 0 to 27	“How often have you felt affected by the following symptoms over the past 2 weeks? e.g., Little interest and pleasure in doing things”	0.86	0.70
Self-identification of having a mental illness	*SELF-I* [39]	5 items, 1 = “don’t agree at all” to 5 = “agree completely”	“My current symptoms could be early signs of a mental disorder.”	0.91	0.87
Attitudes towards seeking psychological help	German version of the *ATSPPH-SF* [40]	10 items, 4-point Likert scale from 1 = “strongly disagree” to 4 = “strongly agree”	“Emotional difficulties like many things, tend to work out by themselves“	0.77	0.83

Note. *α*_sample 1_ internal consistency based on sample of phase 1 *α*_sample 2_ internal consistency based on sample of phase 2. Social distance scale and ERMIS only assessed in phase 2

### Statistical analyses

To assess normal distribution, Shapiro-Wilk tests were conducted on item-level, with significant results (*p *< 0.001). Therefore, non-parametric Spearman correlations (*r*) were calculated for the items with each other and with stigma- and health-related variables. In phases 1 and 2, the interpretation of the *r* refers to Cohen, 1988 [41], small *r* ≥ .10, moderate *r* ≥ .30, and strong correlation *r* ≥ .50. Internal consistency was calculated for the subscales and total scales via Cronbach’s *α.* The analyses of phases 1 and 2 have been carried out with R [42], package *lavaan* [43] and SPSS [44]. The significance level was set at *α* = 0.05 (two-tailed).

### Results - phase 1

#### Sample

Table 2 presents the characteristics of the convenience sample of phase 1 (and the respective information relevant for phase 2).

**Table 2:** Sample descriptives of convenience sample (phase 1) and sample with depressive symptoms (phase 2)

	Phase 1	Phase 2
	*N (%)*,* M (SD)*	*N (%)*,* M (SD)*
*N*	227	1’375
Age	31.9 (11.65)	42.3 (15.24).
Sex		
Female	183 (80.6%)	897 (65.2%)
Male	38 (16.7%)	471 (34.3%)
Diverse	6 (2.6%)	7 (0.5%)
Education		
12/13 years	210 (92.5%)	723 (52.6%)
10 years	13 (5.7%)	466 (33.9%)
9 years	1 (0.4%)	155 (11.3%)
others (a. o. students)	3 (1.3%)	31 (2.3%)
Income		
<1000€	176 (64.3%)	493 (35.9%)
1000–2000€	61 (26.9%)	500 (36.4%)
>2000€	20 (8.8%)	381 (27.7%)
Depression severity	9.17 (5.99)	13.01 (4.21)

Note. Phases 1 and 2 present the two parts of this study to develop the continuum beliefs scale. N = sample size. M = mean score. SD = standard deviation. Income shows monthly income. Income in phase 2 with *n* = 1 missing value. Depression severity assessed via Patient Health Questionnaire (PHQ-9) with sum score 10–14 interpreted as moderate symptom severity (none/minimal 0–4, mild 5–9, moderate 10–14, severe 15–19, extremely severe 20–27) [38]

#### Correlations, internal consistency

Regarding the remaining 16 items considered suitable for scale development, the items assigned to the *State* facet (*α* = 0.79) exhibit moderate to strong correlations among themselves (.318 to .595). Notably, the item 17, „Given extreme circumstances, many of us could show signs of depression“ [31], is negatively correlated with discrimination and blame, while no significant associations with stigma or health-related variables are observed elsewhere.

Regarding the items assigned to the *Person* facet (*α* = 0.74), the correlations among them vary from weak (.158) to strong (.545). However, these items are negatively associated with the agreement with discriminatory statements, guilt, and shame, and agreement with stereotypes. Two items also exhibited this association concerning awareness of stereotypes. Higher agreement to the* Person *items was correlated with better health status, lower depression severity, and more favourable attitudes toward help-seeking (mostly small correlations).

The items assigned to the *Concept* aspect (*α* = 0.69) exhibit moderate to strong correlations among themselves (*r* = .328 to .548). No connection is observed with other health-related variables. Higher agreement with the continuum concept is associated with lower agreement with discrimination statements and stereotypes, and reduced blame. All correlations can be found in the supplement tables S1.2.

#### Item choice

A first item reduction was already made based on the content analyses, balancing the subscales and reducing redundancy of items. From the remaining 16 items, four more items were excluded (items 3, 4, 5, 8, see table S1.3), supported by the aim to achieve a consistent set of three items per subscale, unrelated to a vignette character, and to reduce content-wise redundancy. These exclusions were informed by the characteristics of the items. Item 3 was excluded for reasons of redundancy, and had very small correlations with stigma- and health-related measures (see table S1.2a). Item 4 did not load substantially on any of the exploratory factors (see table S1.3). Item 5 was excluded because of its redundancy with item 18, and a vignette-unrelated variant was preferred. Item 8 had lowest inter-item correlations within the *Person* facet (see table S1.2).

Items were chosen to be generally formulated, without reference to specific vignettes, ensuring the scale’s applicability across contexts without reliance on particular scenarios. However, to encourage further personal involvement, an optional vignette-specific item was introduced for each subscale, mentioning the vignette character’s name. This allowed participants to express their continuum beliefs through personalized narratives (see Supplement S1.1). As a result, an item for the Concept subscale had to be reformulated from existing items, as there was no vignette-specific item associated with this subscale. The resulting *Continuum Beliefs of Mental Illness Scale (CB-MIS)* consists of 12 items (see supplement, table S1.4) with an internal consistency of *α* = 0.79 (*State*: *α* = 0.78; *Person*: *α* = 0.77; *Concept*: *α* = 0.68). A 9-item version excluding the vignette-specific items, demonstrates internal consistency of *α* = 0.76 (*State*: *α* = 0.75; *Person*: *α* = 0.72; *Concept*: *α* = 0.70).

The original included items were scaled from 1–4, 1–5, 1–6, or 1–7 in any case assessing the agreement to the certain phrases (“strongly agree” to “strongly disagree”; or reversed). For the new measure, a 5-point Likert scale ranging from 1 (strongly disagree) to 5 (strongly agree) was chosen. This scale aligns with the majority of the included questionnaires, supports the goal of developing a functional measure, and adheres to recommendations for reliable scaling [45, 46]. This newly developed scale is the basis for the second phase, in which the scale is tested for the first time in a sample of people with depressive symptoms.

## Phase 2: Validation of continuum beliefs Scale

This second phase aims to apply the developed *Continuum Beliefs of Mental Illness Scale (CB-MIS)* to a sample of participants who self-report symptoms of depression and validate the questionnaire.

### Methods – phase 2

#### Scale refinement and qualitative validation

Building upon the item selection conducted in phase 1, items 1, 2, 5 [retrieved from 22 and 29] were rephrased from being depression-specific to refer to general mental health, as the other items originally already did. The scale was qualitatively validated with a sample of *N* = 15 individuals reporting depressive symptoms through cognitive interviews. Those who participated were compensated with vouchers worth 25€ for a health food store. The interviews were carried out in a semi-structured format, guided by an cognitive interview protocol devised by the project team [47]. Alongside the intervention material and various other questionnaires, the items concerning continuum belief items were introduced, asking for participants’ associations and understanding. Techniques of cognitive pretesting were utilized, including probing, which means to ask for a more detailed explanation of the given response [47].

The interview protocol sections addressing continuum beliefs are available as supplement S2.1. In the interviews, it became evident that referencing a familiar individual was apparently pertinent, with recurrent reliance on a person the interviewees knew or on the vignette character. Participants found items in the *State* scale easy to answer, but those in the *Concept* scale challenging, particularly regarding “fundamental differentness,” where nuanced responses were desired. Overall, the term “normal” evoked broader questions on “What/Who is normal?”. The notion that individuals with mental illness “are no normal people” was perceived as hurtful and deemed not to be endorsed without further explanations. Moreover, the distinction between the normalcy of a person with mental health issues and the detached condition was articulated by one interviewee: “Of course, [vignette character] is a perfectly normal person; I would view it differently if it’s not [vignette character] as a person seen as abnormal, but rather the condition seen as abnormal.” Phrasings that were difficult to understand or potentially offensive were reviewed by the authors, with corrections made as necessary.

#### Survey procedure and sample

Based on the study protocol [48], the data collection was conducted through the online panel “respondi AG” (01–09/2021), where individuals are registered to participate in studies by different stakeholders. Participants were invited via email and incentivized (5.75€ maximum) according to standard procedures. A baseline assessment with intervention, and two follow-ups after three and six months were implemented. For details, see the study protocol [48]. The baseline was divided into two parts, comprising the pre-intervention assessment followed by the intervention with the post-intervention questionnaire 36 h later. The intervention involved a symptom vignette featuring a fictional character describing depressive symptoms. Subsequently, varying brief educative manipulations were presented based on a fractional factorial design [49]. This means that subgroups of the sample received different combinations of manipulations, among them a continuum beliefs text or video. See supplement S2.1 for vignette and continuum beliefs intervention.

From the pool of active panellists, a sample of German-speaking adults (*N* = 10’348) was screened for eligibility. Only participants with at least mild depressive symptoms (PHQ-9 sum score ≥ 8, 38) and no current treatment for their mental health problems were included (*N* = 2’132). After exclusions due to data quality (speeders, monotonous answers, attention checks), the sample consisted of *N* = 1’375. A sample of *N* = 984 after three and *N* = 830 after six months participated in the follow-ups.

#### Measures

Table 1 shows the questionnaires measuring stigma and health attitudes. Except for the sum score of depression severity, and the 1-item measure for general health status, the measures have been used as mean scores. Furthermore, age, sex, income, and education (as described for phase 1) were assessed at baseline.

#### Statistical analyses

Shapiro-Wilk revealed non-normal distribution. Within the manuscript, data of the 12-item version are reported, though the analyses have been conducted for the 9-item version as well (see supplement S2). Item descriptives and Spearman correlations are calculated. To assess construct validity, the datasets (respectively for pre- and post-intervention data) were randomly split to create two subsamples.

An exploratory factor analysis (EFA) was conducted as sensitivity analyses to the theoretical foundation. Requirements were tested with the Kaiser-Meyer-Olkin (KMO) measure and the Bartlett test. The number of factors was determined with Eigenvalues (> 1), a scree-plot and Parallel analysis. EFAs were run using Principal Axis Factoring with promax rotation. An oblique rotation method was chosen to allow correlation between the factors as they are all conceptualized as parts of one concept [50].

A confirmatory factor analysis (CFA) was subsequently performed with the other random sub-samples. The items were assigned to the three subscales, which in-turn were amalgamated as a higher-order factor “continuum beliefs”. To address the differences in inclusiveness of the item formulations, items that emphasized differences and included “them”- formulations were modelled as an additional factor. The „MLM“estimator was chosen to account for non-normality and skewness, referring to Maximum Likelihood estimation with robust standard errors and Satorra-Bentler scaled test statistics. The interpretation of goodness-of-fit indices adhered to widely-used conventional cut-off values, as outlined by Hu and Bentler, 1999 [51]: Comparative Fit Index (CFI ≥ 0.95), Tucker-Lewis Index (TLI ≥ 0.95), Root Mean Square Error of Approximation (RMSEA ≤ 0.06) and Standardized Root Mean Residual (SRMR ≤ 0.08;≤0.05 = good [52]). Due to sensitivity of significance to sample size, Chi-square (χ^2^) is evaluated as the ratio of χ^2^to degree of freedom (χ^2^ /df), with values smaller than 2 [53] to 4 [54] indicating acceptable fit. Internal consistency was calculated via Cronbach’s *α* and additional McDonald’s omega (*h)* for each time point. Test-retest reliability was calculated for the scale without vignette-related items (9 item-version) via intra-class correlation (ICC) for the subgroup who did not receive a continuum beliefs intervention across all four time points (*N* = 272). Spearman’s rank correlations coefficients (*r*) of the new scale, subscale and items with a broad range of variables indicate criterion validity.

### Results – phase 2

#### Sample

Table 2 presents the characteristics of the sample with depressive symptoms. The majority of the sample reported moderate depressive symptoms with a PHQ-9 mean score of 13.01 [38].

#### Description of subscales and item analysis

Test-retest-reliability of the nine-item version was good, *ICC* = 0.89 (95%-CI: 0.86;90). The overall scale has an internal consistency of *α* = 0.68 (*h* = 0.61), which is below an interpretation as acceptable (*α* = 0.70). The subscales comprise *α* = 0.72 (*h* = 0.72) for *State*, *α* = 0.63 (*h* = 0.68) for *Person*, and *α* = 0.60 (*h* = 0.62) for *Concept*. Therefore, only the *State* subscale of the nine-item version can be interpreted as acceptable.

The following results refer to the 12-item scale version based on the baseline post-intervention data (see table 3 and S2.1, table S2.5. Inter-item correlations, table S2.6 EFA, table S2.7 item correlations with stigma and other variables). The equivalent results for the nine-item version with pre-intervention data can be found in the Supplement: table S2.3 (item characteristics), table S2.4 Inter-item correlations, table S2.6 EFA and CFA, table S2.7 item correlations with stigma and other variables. Table S2.9 shows correlations of the two scale versions with other stigma and health-related variables.

The 12-item scale overall has an internal consistency of *α* = 0.79 (*h* = 0.79), which can be interpreted as acceptable. The subscales comprise *α* = 0.78 (*h* = 0.78) for *State*, *α* = 0.70 (*h* = 0.72) for *Person*, and *α* = 0.70 (*h* = 0.72) for *Concept*. Regarding the descriptive data in table 3, generally high mean values are observed, indicating a tendency towards higher agreement with the continuum model (“ceiling effect”). The skewness and kurtosis values are within an acceptable range while suggesting non-normally and right-skewed distributions. The item-total correlations are satisfactory (*r* ≥ .30).

**Table 3:** Descriptive statistics of the continuum beliefs scale items at baseline (*N* = 1’375)

Items		*M*		*SD*		Skewness	Kurtosis	Item-total *r*	CFA
**Normality of State of Mental Health Problems**									
1	Most of us from time to time show symptoms of mental illness.	3.80		0.92		-0.48	-0.05	0.60	0.679
2	Given extreme circumstances, many of us could show signs of mental health problems.	4.17		0.84		-0.96	1.01	0.58	0.701
3	Everyone experiences mental health problems at some point; it’s just a matter of how severe these problems are.	4.00		0.95		-0.80	0.23	0.62	0.743
**Normality of Individuals with Mental Health Problems**									
4	Someone with arthritis or a broken leg has just one thing wrong with them, but a person with mental illness is fundamentally different from other people.	3.32		1.30		-0.22	-1.05	0.48	0.283
5	People who have a mental illness are fundamentally different from ordinary people.	3.93		1.09		-0.77	-0.20	0.63	0.573
6	People with mental health problems are normal people just like everyone else.	4.25		0.94		-1.25	1.22	0.34	0.657
**Nosological Concept**									
7	There is a fluid transition between mental health and mental illness.	3.72		1.02		-0.45	-0.23	0.38	0.668
8	Mental health and mental illness are separate and entirely different states.	3.56		1.15		-0.36	-0.59	0.56	0.606
9	There is a clear boundary between being mentally ill and being mentally healthy.	3.62		1.16		-0.45	-0.59	0.59	0.605
**Optional Vignette-Related Items**									
10	Basically, we are all sometimes like [person X]. It’s just a question how pronounced this state is.	4.06		0.93		-0.88	0.47	0.51	0.597
11	There is something about [person X] that makes them fundamentally different from other people.	3.95		1.08		-0.76	-0.23	0.53	0.534
12	[Person X] can be either entirely mentally ill or mentally healthy - there is nothing in between.	4.30		1.01		-1.34	1.03	0.41	0.391

Note. *M* = mean score. *SD* = standard deviation. *r* = item total correlation of items with the respective subscale. Rating on a Likert scale from 1 (strongly disagree) to 5 (strongly agree)

In table S2.4, the correlations among the post-intervention items are presented. Clusters of moderate correlations are evident, aligning with each of the three subscales. Notably, most items show small correlations with items outside the same subscale, but moderate correlations are observed, forming clusters of items independent of the subscales (items 4, 5, 8, 9, 11, 12). These have a substantial similarity on content level, comprising describing a perceived differentness perspective with formulations emphasizing differences, positioning people with mental illness as “the others” (see table S2.1).

#### Factor analyses

Results of EFA have been conducted as sensitivity analyses to the theoretical foundation and can be found in Table S2.5. Regarding CFA, the chi-square statistic yielded *χ²/df* = 150.741, *p* < .001, with *χ²/df* ratio = 3.43 indicating acceptable fit. The sample-corrected *CFI* = 0.940, *TLI* = 0.910; robust *CFI* = 0.935, and *TLI* = 0.903 fall below the recommended cut-off but still exhibit relatively high values. The *RMSEA* = 0.059 (robust = 0.068) and *SRMR* = 0.049 indicate good fit. Based on these indices, the model is considered fitting the data with acceptable, yet not excellent model fit.

The loadings of the twelve items on their respective factors (table 3) are all significant, *p* < .05. While most items load > 0.40 on their respective factors, items 4 and 12 exhibit weaker loadings < 0.40 on the subscales of *Person* and *Concept*. The three factors, *State* (loading = 0.775), *Person* (loading = 0.694), and *Concept* (loading = 0.679), each demonstrate strong loadings on the higher-order factor. Additionally, the items describing perceived differences and outside perspective load each > 0.60 on their factor. This factor is negatively correlated with the higher order factor of continuum beliefs (*r *= -.41).

Table S2.5 contains the CFA of the 9-item scale. The model fit of this CFA relying on the same modelling was even better with *χ²/df* ratio = 2.60. The *CFI* = 0.970, *TLI* = 0.943; sample-corrected, robust *CFI* = 0.973, and *TLI* = 0.949, *RMSEA* = 0.048 (robust = 0.051) and *SRMR* = 0.039.

#### Correlations with stigma

As depicted in table 4, the overall *CB-MIS* exhibits low to moderate correlations with stigma in the 9-item as well as in the 12-item version (see supplement table S2.9). The correlations in the 9-item version are overall lower (up to *r* = .10) than in the 12-item version. Higher agreement to continuum beliefs corresponds to lower agreement with stereotypes, discrimination, shame, social distance, blame, fear, and anger. Pro-social reactions demonstrate small positive correlations, along with an almost negligible association with awareness of stereotypes. Upon examining the subscales, all three show similar associations, yet the *Person* subscale displays the most pronounced moderate correlations for the 12-item version. Specifically, concerning stereotype agreement, *r*=-.46 is noteworthy. Positive correlations are observed with help-seeking attitudes and self-identification of having a mental illness. The correlations of single items can be found in the supplement table S2.6 for time point 1 and table S2.7 for time point 2.

**Table 4:** Correlations of continuum beliefs with stigma and health-related variables at time point 2 post-intervention

	Subscales				
	Overall scale	State	Person	Concept	Item 10^a^
Stereotype awareness	0.069^*^	0.128^**^	− 0.019	0.050	0.081^**^
Stereotype agreement	**− 0.398** ^**^	− 0.154^**^	**− 0.462** ^**^	**− 0.319** ^**^	− 0.124^**^
Discrimination	**− 0.354** ^**^	− 0.144^**^	**− 0.380** ^**^	− 0.291^**^	− 0.104^**^
Shame	− 0.206^**^	− 0.161^**^	− 0.193^**^	− 0.125^**^	− 0.072^**^
Social distance	**− 0.384** ^**^	− 0.194^**^	− 0.394^**^	− 0.265^**^	− 0.115^**^
Blame	**− 0.376** ^**^	− 0.173^**^	**− 0.386** ^**^	**− 0.327** ^**^	− 0.095^**^
Fear	− 0.288^**^	− 0.150^**^	**− 0.346** ^**^	− 0.218^**^	− 0.092^**^
Anger	− 0.299^**^	− 0.152^**^	**− 0.337** ^**^	− 0.249^**^	− 0.124^**^
Pro-social reactions	0.142^**^	0.199^**^	0.082^**^	0.077^**^	0.164^**^
Depression severity	− 0.012	0.040	− 0.050	− 0.055^*^	− 0.019
Self-identification	0.211^**^	0.160^**^	0.118^**^	0.178^**^	0.072^**^
HS Attitudes	**0.320** ^**^	0.214^**^	0.230^**^	0.257^**^	0.126^**^

Note. Spearman correlations *r*. In bold ≥ 0.30. ^a ^Item 10: “Basically, we are all sometimes like [person X]. It’s just a question how pronounced this state is.”, for comparison. * indicates *p* < .05. ** indicates *p* < .01

#### Convergent and divergent validity

To compare the new scale with established measurement methods, the most used item in research to date [20] “Basically, we are all sometimes like [person X]. It’s just a question how pronounced this state is.” (number 10 in *CB-MIS*) by Schomerus and colleagues [15] was compared to the *CB-MIS*. Item 10 is strongly positive correlated with the subscale *State* (*r* = .746, *p* < .01), and shows small positive correlations with *Person* (*r* = .206, *p* < .01) and *Concept* (*r* = .203, *p* < .01). Compared to the 9-item version at time point 1, not including item 10, there is a moderate positive correlation with *State* (*r* = .440, *p* < .01), and still small positive associations with *Person* (*r* = .179, *p* < .01) and *Concept* (*r* = .130, *p* < .01).

As shown in table 3, item 10 has similar associations compared to the *State* subscale, correlating small negative with stigma measures (or small positive with stereotype awareness and pro-social reactions). However, regarding the health-related variables, item 10 appears to be less responsive. It exhibits a near-zero correlation with depression severity, self-identification, and a smaller correlation than the *State* subscale with help-seeking attitudes. *Person* and *Concept* facets more strongly depict the negative association with stigma and serve as a further addition to the existing assessment.

## Discussion

To advance the continuum belief assessment deemed urgently necessary in mental health research, due to a current lack of psychometric validation and reliance on limited vignette-based or single-item assessments [20]. Previous research has tended to overlook the multifaceted nature of continuum beliefs, which encompass both conceptual (e.g., nosological) and experiential (e.g., personal experiences with individuals affected by mental health issues) dimensions. The newly developed *Continuum Beliefs of Mental Illness Scale (CB-MIS)* introduces important innovations in terms of its multifactorial assessment, generalizability, and validity, all of which are discussed in the following sections.

Firstly, the scale is routed in an in-depth examination of the research landscape [17, 20], developed in a theory-driven manner encompassing different inherent facets of continuum beliefs and contra points to essentialist beliefs [16] in a multifactorial scale. The three subscales were derived from Tomczyk et al. [20]. Item selection was guided by independent ratings and expert discussions at a content level. Principal factor analysis validated the item assignment at the data level, while internal consistencies and correlations were used to assess reliability.

The subscales delineate distinct frames of reference for conceptualizing mental health and illness along a continuum of abstraction, increasing in subjective complexity, as reported by participants in the cognitive interviews of this study. Thus, the subscales encompass levels of abstraction, beginning with an experience-oriented symptom state, followed by the subjective normality of individuals with mental health problems, which reflects a person-centered frame of reference, and concluding with the fundamental conceptualization of mental health and illness. The initial levels are more conceptually accessible to respondents and may be influenced by personal contact experiences [25], which can be further assessed using the new scale. The illness concept, however, is less connected to personal encounters and more aligned with broader social categorization. Additionally, this concept likely underpins the entire model, similar to essentialist beliefs [16], and connects to stigma, where social categorization fosters discrimination against out-group members [55].

Furthermore, the *CB-MIS* was compared to the single-item measure [15], which produced results similar to those of the *State* subscale, although potential confounding arises due to its inclusion as a vignette-related item within the subscale. These findings serve as an initial step for future comprehensive analyses of convergent and divergent validity. Interestingly, the single item contains both the reference-frames of *State* and *Person* showing small negative correlations with stigma over various studies [17]. The new scale differentiates these aspects. The *Person* subscale plays a significant role in its associations with stigma. Participants who disagreed more with the idea of individuals with mental illnesses being “fundamentally different” referring to an idea of normality/non-normality also displayed lower levels of stigma across all measures. This correspondence may stem from a shared underlying cognitive framework that involves social identity and reflections of in-group and out-group [55], closely linked to perceptions of deviation from a particular “norm,” thus influencing attitudes towards individuals [8, 31, 56]. Similar patterns of significant associations were observed for the *State* and *Concept* subscales, although with lower values. Hence, the scale aligns with anticipated responses to stigmatization based on prior research [17], and enhances it by encompassing a range of stigma attitudes and a sample exhibiting depressive symptoms.

A predetermined objective was to develop a vignette-independent scale, thereby reducing its reliance on specific study materials [18], and potentially enhancing its objectivity in measuring continuum perceptions. Nevertheless, attention was given to including items that facilitate a connection to personal experience during the development process, thereby engaging respondents in expressing their agreement with statements about the continuum of health and illness [17]. In Phase 2, each subscale included a vignette-based item as an optional variant to enhance the scale’s flexibility and effectiveness. Moreover, the vignette-item 10 (and also 1) was among the most frequently used items in previous research across a variety of contexts and mental health conditions [20]. The correlation matrix indicates that items 10 and 11 primarily correlate with the respective subscales. However, item 12, which was assigned to the *Concept* subscale to ensure an even distribution of items, loaded comparably on both the *Person* and *Concept* subscales. This could be attributed to its dual focus, incorporating a person-centered perspective (emphasizing the normality of individuals with mental illness) alongside a conceptual argument regarding the mental health-illness continuum (nosological framework).

Respondent involvement could also be fostered by minimizing the perceived distance of respondents to the continuum topic, as suggested by the item formulations. Emphasizing perceived similarities [24], or describing a continuum that demands self-positioning of the respondent might promote an in-group perspective [55]. This approach contrasts with items that require engagement with an outside perspective or emphasize differences, which do not necessitate self-positioning. This differentiation was applicable to all but four items from the item pool which exclusively focused on symptom descriptions [23]. Notably, in the *CB-MIS*, respondent-inclusive formulations are primarily found within the *State* subscale, whereas the *Person* and *Concept* subscales predominantly feature items framed from an outside perspective. Future studies could pay closer attention to different associations with stigma emerging from items of these classification categories, particularly given their confoundation with the subscales in the current scale design. Thus, in this study, the issue was addressed by grouping all items employing “differentness” or “othering” formulations and incorporating a factor negatively associated with the higher-order factor of continuum beliefs. Apart from that, the respondent-including and similarity-enhancing items could not be represented in the factor analysis. Including this factor would have been redundant with the higher-order factor. Given that this aspect was only indirectly explored in the current study, future research should aim to examine it more thoroughly. This could involve the use of implicit measures [e.g. 57], and the development of a distinct scale to assess the relationships between the “like us” and “like others” poles and other relevant variables. Such efforts may reveal variations based on factors such as social desirability, educational attainment, or levels of personal experience.

Regarding the content of the items, the item 4 (“Someone with arthritis or a broken leg has just one thing wrong with them, but a person with mental illness is fundamentally different from other people.”) builds an exception with surprisingly low loading on the Person subscale. The fitting might be influenced by the framing of physical disability which is missing in all other items of this subscale. Multiple comments from the preliminary interviews described this item as misleading and challenging to comprehend. Both conditions do elicit self-stigma [58], which was not controlled for, potentially limiting the item’s discriminative capacity. Given efforts to integrate the care of physical and mental health conditions [59], the question of arises, whether this should be accompanied by targeted education or anti-stigma messaging to foster understanding and ensure that all affected individuals feel acknowledged and supported.

The internal consistency of the scale with 12 items (*α* = 0.79) can be interpreted as acceptable. The subscales perform just as well with internal consistencies of *α* = 0.78 (*State*), *α* = 0.70 (*Person*), and *α* = 0.70 (*Concept*). The 9-item version, with α = 0.68 is slightly less than acceptable, *α* = 0.72 (*State*), *α* = 0.63 (*Person*), and *α* = 0.60 (*Concept*). However, the 9-item version demonstrates good test-retest reliability over a period of 6 months (ICC = 0.89). In phase 2, construct validity was examined in separate split-half samples. The situation reverses here, with the 9-item version providing a better, very good model fit, while the 12-item version is deemed interpretable. Consequently, the *CB-MIS* with three subscales and their corresponding items emerges as valid both in exploratory and confirmatory factor analyses.

The variant with nine items has even been employed within an intervention study [60], with investigations stemming from the same dataset as these validation analyses. A significant increase in agreement with overall continuum beliefs was observed following the continuum beliefs text intervention. This finding underscores the change sensitivity of the *CB-MIS* (9 items). Future studies could build on this by exploring the potential of incorporating vignette-related items to further enhance the scale’s applicability and depth of analysis.

The *CB-MIS* has several implications. For stigma interventions, researching the subscales and their determinants and influences [e.g., 61], as well as their relevance for different subgroups is advisable. Such subgroups could comprise different mental illnesses and illness variables like symptom severity [26] or problem recognition [62] as well as demographic variables, like socioeconomic status and milieu factors [63]. Moreover different interventional approaches might alter different parts of continuum beliefs, with cognitive strategies, like knowledge given from an expert [24], potentially having a special influence on the nosological concept. Social and experience-based strategies like meeting a person with lived experience [30], or tailored video interventions [64] might especially affect agreement with normality of individuals, while affective-emotional strategies might target state normality. Depending on personality and culture [65], values, experiences, or health, individuals may respond differently to these approaches [16]. These diverse intervention approaches, could be accurately and economically reflected with the new scale, making it an attractive tool giving new potential to intervention research on altering stigma through continuum beliefs.

Based on exploratory findings, continuum beliefs can also foster help-seeking attitudes, for instance by leading to malleability beliefs regarding mental health, allowing for more positive assessments of controllability and self-efficacy, and creating positive social norms [66]. Although this study did not examine all of these associations, they represent clear directions for future research on continuum-based interventions.

### Strengths & limitations

This scale represents the first psychometrically validated tool to assess multiple facets of continuum beliefs, showing acceptable model fit and strong item loadings on their respective subscales. We consider our approach to scale development a significant strength. It began with content ratings and expert discussions based on theoretical frameworks in a convenience sample, followed by questionnaire adaptation, qualitative evaluation of participant reactions and comprehensibility, and quantitative validation in a comparatively large symptomatic sample. However, this approach also has drawbacks regarding standardization and the fact that in phase 1, item selection was not primarily based on psychometric criteria but on content which can be biased by subjective assessments. Data collection was conducted online based on self-reports, which introduces the potential for bias from motivated participants and social desirability effects. A limitation is that the scale development occurred within a sample that included an intervention design, meaning that not all data could be used for the analysis of intraclass correlation for test-retest reliability. Furthermore, the vignette-related items were assessed at only one time point, therefore we were not able to calculate test-retest-reliability for the 12-item version.

The scale validation in phase 2 was conducted in a sample with depressive symptoms, potentially reducing comparability with other studies. However, scale development does not solely rely on this sample, and one can assume that attitudes and beliefs of the public are internalized by individuals who are themselves affected [32]. Still, mental illness contemplation might be prominent in this sample. The scale’s association with depressive symptom severity implied close to zero correlations with the continuum model. Hence, we assume that the scale is effective not only in samples exhibiting depressive symptoms but also in convenience samples. Subsequent studies could focus on further psychometric refinements out of scope in our work but pivotal for establishing construct validity. These include tests for measurement invariance via multigroup confirmatory factor analysis (CFA), known-groups validity, and further investigations to test the scales applicability in different samples. Nevertheless, it would be valuable to replicate the analyses in other samples of individuals with depressive symptoms to account for potential bias, as our sample consisted of individuals not currently undergoing treatment, who may not have a formal diagnosis, and who were asked to complete a cognitively demanding survey.

Another strength is the involvement of input from individuals affected by depressive symptoms. Interviews were conducted with them, presenting the developed items and eliciting their associations, feelings, and understanding. We are aware that the participatory approach in this scale development is still rudimentary, and future studies would benefit from collaborating with individuals with lived experience and them leading research [67].

Additionally, the study lacks generalizability for different mental illnesses and minority groups. A convenience sample (phase 1) and a sample of people with depressive symptoms (phase 2) were examined without oversampling or stratification regarding specific dimensions of inequity (e.g., gender, race/ethnicity). Special attention should be given to these particular groups and to all others experiencing structural discrimination due to their identity in the healthcare system [68, 69]. It would be crucial for future studies to focus on groups disadvantaged due to multiple characteristics, as stigma and the associated burden compound in such populations [70, 71].

## Conclusion

This study describes the development and validation of the *Continuum Beliefs of Mental Illness Scale (CB-MIS)* for the multifactorial assessment of continuum beliefs of mental health and illness. The inherent facets of the continuum model - *State*, *Person* and *Concept* - corresponding to “normality of being in a state of having mental health problems and symptoms”, “normality of the individuals with mental health problems”, and “nosological concept of mental health and illness”, provide various entry points for further exploring the scale’s associations with other constructs like health-related ones. Furthermore, the scale comprises items that differ in their implied perception of distance between respondents and the continuum model, as well as individuals with mental illness. This is reflected in the item formulations, which either emphasize similarities or differences with those affected and frame a continuum of affected persons being “like us” or “like them/others.” This aspect warrants further exploration in relation to further aspects of conceptualizing the continuum model, and to whether the degree to which respondents are willing to locate themselves on a continuum together with people with mental illness has implications for stigmatization. Ultimately, we found that both versions of the *CB-MIS*, featuring either 9 or 12 items, performed fundamentally similar in terms of factor structure, and correlations with stigma and health-related variables. This is not surprising given their shared items. The 9-item variant, however, already has confirmation through test-retest reliability and change sensitivity. Despite this, the 12-item variant demonstrated stronger internal consistency and correlations with stigma. We recommend further psychometric testing and application of both versions, depending on specific research goals. The 12-item version includes the frequently used item from Schomerus et al., 2013 [15], ensuring comparability with past studies. In sum, the 12-item version of the *CB-MIS* presents an intriguing opportunity, despite having less available validation yet, as it offers greater flexibility and involvement of the participants with the inclusion of a vignette, and therefore might be the best *CB-MIS* version for further research. However, the practical choice between these scales may depend on the specific requirements of the intended study, particularly as the additional three items in the 12-item version relate to a vignette component, which may or may not align with the study’s focus. Subgroup-specific investigations are urgently needed to make the scale universally applicable and to test its result stability across different study designs, mental health conditions, and individual characteristics. As continuum beliefs robustly demonstrate negative correlations with stigma, the possibilities for change through interventions can be explored in detail based on this new scale. This could further advance anti-stigma efforts in this area and explore the potential of the continuum belief model.

## Electronic supplementary material

Below is the link to the electronic supplementary material.

Supplementary Material 1

## Data Availability

The data that support the findings of this study are available on request from the corresponding author. The code behind this analysis has been made publicly available at the Open Science Framework (OSF) and can be accessed at DOI 10.17605/OSF.IO/MYJT7 (https://osf.io/myjt7/?view_only=5a0ef8ca6e0b45999be16abb6e491a9b).
