# Supplementary material for: Psychometric validation of the continuum beliefs of mental illness scale (CB-MIS) and its associations with stigma
Source: BMC Psychiatry. 2025 Feb 4;25:96. doi: 10.1186/s12888-024-06467-8 (PMC11796137; doi:10.1186/s12888-024-06467-8)
Supplement: Supplementary file 1 — Supplementary Material 1 [file 12888_2024_6467_MOESM1_ESM.docx]

**Additional Material: Psychometric Validation of the Continuum Beliefs of Mental Illness Scale (CB-MIS)**

**Supplement Phase 1**

**S1.1 Labelled Symptom Vignette “Alex”**

Instruction:

*Original (German):* „Als Nächstes werden Sie einen kurzen Text einer Person zu lesen bekommen. Lesen Sie diesen aufmerksam durch und machen Sie dann mit der Befragung weiter.“

*Translation (English):* “Next, you'll read a short text from someone. Take a moment to read it carefully, then continue with the survey.”

Vignette:

*Original (German):* „Hallo, ich bin Alex und möchte Ihnen gern von den Erfahrungen erzählen, die ich in letzter Zeit gemacht habe. Seit einigen Monaten fühle ich mich oft traurig und kann gar nicht genau bestimmen, was mich runterzieht. Ich kann keine richtige Freude mehr empfinden und es macht mir nicht so viel Spaß wie sonst, Zeit mit meinen Freunden zu verbringen. An manchen Tagen fängt es morgens schon damit an, dass es mir schwerfällt, kleinste Entscheidungen zu treffen, wie die Wahl der Kleidung oder des Frühstücks. Es fällt mir schwer, mich aufzuraffen, um aus dem Haus zu gehen und ich kann mich auch schlechter auf meine Arbeit konzentrieren. Häufig zerbreche ich mir wegen Kleinigkeiten den Kopf und mache mir für alles Mögliche Vorwürfe. Inzwischen ist mir klar geworden, dass es sich um Anzeichen einer Depression handelt. Ich weiß nicht, ob Sie sich in einer ähnlichen Situation befinden – wenn ja, kann ich Ihnen nur sagen: ich weiß, wie schwer es ist!“

*Translation (English):* “Hey, I'm Alex, and I wanted to share some of the experiences I've been going through lately. For a few months now, I've been feeling pretty down, and I can't quite pinpoint what's bringing me down. I just can't seem to find real joy, and hanging out with my friends isn't as fun as it used to be. Some days, it starts in the morning when even the smallest decisions, like what to wear or what to have for breakfast, feel challenging. Getting myself out of the house becomes a struggle, and I find it harder to focus on my work. I often overthink the little things and end up blaming myself for all sorts of stuff. I've come to realize that these are signs of depression. I don't know if you're going through something similar, but if you are, I just want to say: I get how tough it can be!”

**Table S1.1**

*Overview of Item Pool and Content Ratings*

|  |  |  |  |  |  |
| --- | --- | --- | --- | --- | --- |
|  |  |  | Content Classification | |  |
| Authors, Name, Scaling | Items English/German | # | Facet | Implied Distance |  |
| **Schomerus et al., 2013,** Belief in a continuum of symptom experience | **Basically we are all sometimes like this person, It’s just a question how pronounced this state is. / Im Grunde sind wir alle manchmal wie Alex. Es ist nur eine Frage, wie ausgeprägt dieser Zustand ist.** | 1 | State | Inside Perspective, Similarity | |
| **Schomerus et al., 2016,** Continuity beliefs and fundamental difference, (scale 1-5: 1= strong agreement;  5= strong disagreement) | Sometimes we are all at least a little like Alex, it is only a question of how pronounced this state is. / Im Grunde sind wir alle manchmal wie Alex. Es ist nur eine Frage, wie ausgeprägt dieser Zustand ist. | 2 | State | Person, Inside Perspective, Similarity | |
|  | To some extent, most persons will experience problems that are similar to those of Alex. / In gewissem Ausmaß werden die meisten Menschen psychische Probleme erleben, die den Problemen von Alex ähnlich sind. | 3 | State | Person, Outside Perspective, Similarity | |
|  | Overall, Alex‘ problems are abnormal. / Alles in allem sind Alex' psychische Probleme unnormal. | 4 | Person | Person, Differentness | |
|  | People with problems like Alex are normal persons like everybody else. / Menschen mit psychischen Problemen wie Alex sind normale Menschen wie alle anderen auch. | 5 | Person | Person, Outside Perspective, Similarity | |
|  | **There is something about Alex that makes her fundamentally different from other people. / Es gibt etwas an Alex, das sie grundlegend verschieden von anderen Menschen macht.** | 6 | Person | Person, Outside Perspective, Differentness | |
|  | **Someone with arthritis or a broken leg has just one thing wrong with them, but a person with mental illness (original: like Anne) is fundamentally different from other people. / Bei einer Person mit Gelenkschmerzen oder einem Armbruch ist nur eine Sache nicht Ordnung, aber ein Mensch mit psychischen Problemen ist grundlegend verschieden von anderen Menschen.** | 7 | Person | Outside Perspective, Differentness | |
|  | Alex is in a state of mind that normal persons simply cannot understand. / Alex ist in einem Zustand, den normale Menschen einfach nicht verstehen können. | 8 | Person | Person, Differentness | |
| **Thibodeau et al., 2017,** Continuum and categorical beliefs, (scale 1-5:  rating not specified) | **People who have depression are fundamentally different from ordinary people. / Menschen mit Depression sind grundlegend anders als gewöhnliche Menschen.** | 9 | Person | Outside Perspective, Differentness | |
|  | **People who have depression (original: schizophrenia) have symptoms [low mood, lack of motivation] (original: [delusions, hallucinations]) that are similar to the occasional experiences of ordinary people. /** **Menschen mit Depression haben Symptome (Niedergeschlagenheit, Antriebslosigkeit), die gelegentlichen Erfahrungen von gewöhnlichen Menschen ähneln.** | 10 | State | Outside Perspective, Similarity | |
| **Thibodeau et al., 2018,** Endorsement continuum & categorial belief, (scale 1-4:  1=strongly disagree; 4= strongly agree) | Even people without depression (original: schizophrenia) occasionally experience low moods and a lack of motivation. / Selbst Menschen ohne Depression erleben gelegentlich Niedergeschlagenheit und Antriebslosigkeit. | 11 | State | Outside Perspective, Similarity | |
|  | **Depression (original: schizophrenia) is an "either/or" phenomenon – there is nothing in between. / Depression ist ein “Entweder-Oder“-Phänomen – es gibt nichts dazwischen.** | 12 | Concept | Outside Perspective, Differentness | |
|  | Anybody could develop depression (original: schizophrenia) under the right circumstances. / Unter den richtigen Umständen könnte jeder eine Depression entwickeln. | 13 | Person | Inside Perspective, Similarity | |
|  | Symptoms of depression (original: schizophrenia) represent clear departures from the way normal people function. / Symptome der Depression stellen deutliche Abweichungen von der Art und Weise dar, wie normale Menschen funktionieren. | 14 | Sate | Outside Perspective, Differentness | |
| **Norman et al., 2008,** Belief in continuity between everyday experience and illness, (scale 1-5:  1= strongly agree; 5= strongly disagree) | **Most of us from time to time show symptoms of schizophrenia/depression. /** **Ab und zu zeigen die meisten von uns Symptome einer Depression.** | 15 | State | Inside Perspective, Similarity | |
|  | Normal people can have some of the symptoms of schizophrenia/depression. / Normale Menschen können einige Symptome einer Depression haben. | 16 | Person | Outside Perspective, Similarity | |
|  | **Given extreme circumstances, many of us could show signs of schizophrenia/depression. /** **Unter extremen Umständen könnten viele von uns Anzeichen von Depression zeigen.** | 17 | State | Inside Perspective, Similarity | |
| **Unpublished from prior project,** (scale 1-7: 1 = don’t agree at all to; 7 = agree completely) | **People with mental health problems are normal people just like everyone else. /** **Menschen mit psychischen Problemen sind normale Menschen wie alle anderen auch.** | 18 | Person | Outside Perspective, Similarity | |
|  | **There is a fluid transition between mental health and mental illness. /** **Zwischen psychischer Gesundheit und psychischer Krankheit gibt es einen fließenden Übergang.** | 19 | Concept | Outside Perspective, Similarity | |
|  | **Mental health and mental illness are separate and entirely different states. / Psychische Gesundheit und psychische Krankheit sind getrennte und vollkommen unterschiedliche Zustände.** | 20 | Concept | Outside Perspective, Differentness | |
|  | **Everyone experiences mental health problems at some point; it's just a matter of how severe these problems are. /** **Alle Menschen haben irgendwann einmal psychische Probleme, es ist nur die Frage, wie stark diese Probleme sind.** | 21 | State | Inside Perspective, Similarity | |
| **Wiesjahn et al., 2014,** CBQ: Continuum belief questionnaire, (scale 1-6:  1= completely disagree; 6= completely agree) | Experiences in depression (original: schizophrenia) are qualitatively different from normal experiences. / Das Erleben einer Depression unterscheidet sich qualitativ von normalem Erleben. | 22 | State | Outside Perspective, Differentness | |
|  | Low mood, as well as loss of interests and joy (original: Hallucinations or thought disorder) can happen to anyone if they are stressed. / Niedergeschlagenheit und der Verlust von Interesse oder Freude können unter sehr belastenden Bedingungen bei jedem auftreten. | 23 | State | Inside Perspective, Similarity | |
|  | Patients with depression (original: schizophrenia) are very different to other mental health patients. / Patienten mit einer Depression sind sehr anders als Patienten mit anderen psychischen Störungen. | 24 | Person | Outside Perspective, Differentness | |
|  | Many people experience depressive (original: psychotic) symptoms without feeling distressed by them. / Viele Personen erleben depressive Symptome ohne sich dadurch sonderlich belastet zu fühlen. | 25 | State | Outside Perspective, Similarity | |
|  | Sometimes it is not easy to judge whether low mood is truly part of a depression (original: a belief is truly delusional)./ Manchmal ist es schwer zu beurteilen, ob Niedergeschlagenheit wirklich Teil einer Depression ist. | 26 | State | Classification not applicable | |
|  | The low mood or lack of motivation (original: hallucination or delusion) is not as relevant as the distress associated with it. / Es ist weniger relevant, ob eine Person antriebslos ist oder sich niedergeschlagen fühlt, sondern vielmehr, ob sie sich dadurch belastet fühlt. | 27 | State | Classification not applicable | |
|  | Depressive moods (original: Delusions) can be quite understandable. / Depressive Stimmungen können ziemlich nachvollziehbar sein. | 28 | State | Similarity | |
|  | **There is a clear boundary between being mentally ill and being mentally healthy. / Es gibt eine klare Grenze zwischen psychischer Krankheit und psychischer Gesundheit.** | 29 | Concept | Outside Perspective, Differentness | |
|  | Anyone can experience low mood and a lack of motivation (original: a hallucination) now and again. / Jeder kann hin und wieder niedergeschlagen sein und sich antriebslos fühlen. | 30 | State | Inside Perspective, Similarity | |
|  | It is easier to empathize with anxiety problems than with depression (original: delusions). / Es ist leichter für Angstprobleme Verständnis aufzubringen als für Depression. | 31 | State | Outside Perspective, Differentness | |
|  | People with schizophrenia (original: depression) are much easier to understand than those with depression (orig: schizophrenia). / Personen mit Schizophrenie sind viel einfacher zu verstehen als Personen mit Depression. | 32 | State | Outside Perspective, Differentness | |
|  | It is not a question of having depression (original: schizophrenia) or not, but rather a question of the severity of the symptoms. / Es ist weniger relevant, ob man eine Depression hat oder nicht, sondern wie schwer die Symptome sind | 33 | State | Outside Perspective | |
|  | Depression (original: Schizophrenia) is a psychological disorder like any other (e.g., anxiety (not included in original). / Depression ist eine psychische Störung wie jede andere (z. B. Angststörungen) | 34 | State | Classification not applicable | |
|  | People who experience single depressive (original: psychotic) symptoms are not necessarily mentally ill. / Menschen, die einzelne depressive Symptome haben sind nicht notwendigerweise psychisch krank | 35 | State | Outside Perspective | |
|  | Most symptoms of depression (original: schizophrenia) are quite common in the normal population. / Die meisten Symptome einer Depression tauchen auch in der Normalbevölkerung recht häufig auf | 36 | State | Outside Perspective, Similarity | |
|  | Deep sadness, or the loss of interest and motivation (original: Some strongly held religious or political beliefs) are barely distinguishable from delusions. / Tiefe Traurigkeit, der Verlust von Interesse und Antriebslosigkeit sind von Depressionen kaum zu unterscheiden | 37 | State | Classification not applicable | |

*Note*. # Numbers of items. in Bold: Selected items for the scale creation. The items are provided in German with adaptations pertaining to targeting depression. English translations are provided, with reference to the original terminology of schizophrenia in brackets, if the case. Content analysis encompasses marker words, which are assessed to most suitably align with the respective items. Facets include State, Person, or Concept; Aspects of implied distance: Us vs. Them-formulations, Person mentioned; and the aspect of Similarity or Differentness is considered.

**Table S1.2a**

*Correlation Coefficients of the Items assigned to the* ***State*** *Facet (N = 227) with Stigma and Health Variables*

|  | Item 2 | Item 3 | Item 15 | Item 17 | Item21 |
| --- | --- | --- | --- | --- | --- |
| Item 2 | 1.000 |  |  |  |  |
| ~~Item 3~~ | .442^**^ | 1.000 |  |  |  |
| Item 15 | .435^**^ | .438^**^ | 1.000 |  |  |
| Item 17 | .318^**^ | .322^**^ | .595^**^ | 1.000 |  |
| Item 21 | .506^**^ | .307^**^ | .527^**^ | .450^**^ | 1.000 |
| Self-identification of having mental illness | -.180^**^ | -.002 | -.096 | -.002 | -.118 |
| Health Status | .116 | -.002 | .039 | .101 | .114 |
| Depressive Symptom Severity | -.108 | .039 | -.065 | -.017 | -.058 |
| Discrimination | .010 | .035 | -.083 | -.228^**^ | -.040 |
| Blame | -.081 | -.043 | -.129 | -.150^*^ | -.077 |
| Shame | .081 | .094 | .018 | .057 | -.022 |
| Agreement to Stereotypes | .033 | .047 | -.117 | -.110 | .000 |
| Awareness of Stereotypes | -.109 | .024 | -.080 | .043 | -.055 |
| Help-Seeking Attitudes | .020 | -.060 | .061 | .023 | -.006 |

*Note*. Spearman correlation coefficients of items assigned to the facet “state”, normality of being in a state of having mental health problems. Item numbers refer to table S1.1. Items marked with a strikethrough were excluded during the scale development process. * indicates *p* < .05. ** indicates *p* < .01.

**Table S1.2b**

*Correlation Coefficients of the Items assigned to the* ***Person*** *facet (N = 227) with Stigma and Health Variables*

|  | Item 4 | Item 5 | Item 6 | Item 7 | Item 8 | Item 9 | Item 18 |
| --- | --- | --- | --- | --- | --- | --- | --- |
| ~~Item 4~~ | 1.000 |  |  |  |  |  |  |
| ~~Item 5~~ | .285^**^ | 1.000 |  |  |  |  |  |
| Item 6 | .261^**^ | .217^**^ | 1.000 |  |  |  |  |
| Item 7 | .217^**^ | .262^**^ | .492^**^ | 1.000 |  |  |  |
| ~~Item 8~~ | .158^*^ | .093 | .408^**^ | .328^**^ | 1.000 |  |  |
| Item 9 | .194^**^ | .316^**^ | .498^**^ | .545^**^ | .308^**^ | 1.000 |  |
| Item 18 | .273^**^ | .543^**^ | .313^**^ | .351^**^ | .146^*^ | .400^**^ | 1.000 |
| Self-identification of having mental illness | .097 | .052 | .047 | -.057 | -.187^**^ | -.024 | .063 |
| Health Status | .010 | .098 | .036 | .111 | .159^*^ | .171^**^ | .147^*^ |
| Depressive Symptom Severity | -.025 | -.035 | .001 | -.131^*^ | -.160^*^ | -.105 | .018 |
| Discrimination | -.219^**^ | -.079 | -.250^**^ | -.169^*^ | -.141^*^ | -.185^**^ | -.152^*^ |
| Blame | -.147^*^ | -.145^*^ | -.193^**^ | -.188^**^ | -.122 | -.197^**^ | -.250^**^ |
| Shame | -.111 | -.065 | -.081 | -.144^*^ | -.138^*^ | -.225^**^ | -.148^*^ |
| Agreement to Stereotypes | -.117 | -.150^*^ | -.174^**^ | -.208^**^ | -.138^*^ | -.239^**^ | -.307^**^ |
| Awareness of Stereotypes | -.111 | -.060 | -.093 | -.130 | -.199^**^ | -.179^**^ | -.028 |
| Help-Seeking Attitudes | .109 | .224^**^ | .067 | .181^**^ | .086 | .256^**^ | .191^**^ |

*Note*. Spearman correlation coefficients of items assigned to the facet “Person”, normality of individuals with mental health problems. Item numbers refer to table S1.1. Items marked with a strikethrough were excluded during the scale development process. * indicates *p* < .05. ** indicates *p* < .01.

**Table S1.2c**

*Correlation Coefficients of the items assigned to the* ***Concept*** *Facet (N = 227) with Stigma and Health Variables*

|  | Item 12 | Item 19 | Item 20 | Item 29 |
| --- | --- | --- | --- | --- |
| Item 12 | 1.000 |  |  |  |
| Item 19 | .328^**^ | 1.000 |  |  |
| Item 20 | .375^**^ | .518^**^ | 1.000 |  |
| Item 29 | .411^**^ | .493^**^ | .548^**^ | 1.000 |
| Self-identification of having mental illness | .079 | .126 | .068 | .225^**^ |
| Health Status | .100 | -.007 | .027 | -.028 |
| Depressive Symptom Severity | -.016 | .049 | -.043 | .128 |
| Discrimination | -.257^**^ | -.235^**^ | -.265^**^ | -.262^**^ |
| Blame | -.247^**^ | -.175^**^ | -.199^**^ | -.232^**^ |
| Shame | -.123 | -.022 | -.117 | -.064 |
| Agreement to Stereotypes | -.238^**^ | -.179^**^ | -.275^**^ | -.276^**^ |
| Awareness of Stereotypes | -.033 | -.098 | -.117 | -.076 |
| Help-Seeking Attitudes | .090 | .008 | .132^*^ | .038 |

*Note*. Spearman correlation coefficients of items assigned to the facet “Concept”, noslogical cocenpt of a continuum of mental health and illness. Item numbers refer to table S1.1. * indicates *p* < .05. ** indicates *p* < .01.

**Table S1.3**

*Exploratory Factor Analysis of the initial item pool (N=227)*

|  |  | Item total r | 1 | 2 | 3 | 4 |
| --- | --- | --- | --- | --- | --- | --- |
| Item 2 | Basically we are all sometimes like this person, It’s just a question how pronounced this state is. | .46 | **0.64** | -0.11 | -0.14 | 0.20 |
| ~~Item 3~~ | To some extent, most persons will experience problems that are similar to those of Alex. | .40 | **0.57** | -0.09 | 0.09 | 0.00 |
| Item 15 | Most of us from time to time show symptoms of depression. | .54 | **0.77** | 0.16 | 0.05 | -0.20 |
| Item 17 | Given extreme circumstances. many of us could show signs of depression. | .51 | **0.57** | 0.11 | 0.12 | -0.04 |
| Item 21 | Everyone experiences mental health problems at some point; it's just a matter of how severe these problems are. | .44 | **0.73** | -0.02 | -0.08 | 0.00 |
| ~~Item 4~~ | Overall. Alex’ problems are abnormal. | .34 | 0.06 | 0.04 | 0.14 | 0.24 |
| ~~Item 5~~ | People with problems like Alex are normal persons like everybody else. | .49 | -0.02 | -0.08 | -0.12 | **0.95** |
| Item 6 | There is something about Alex that makes her fundamentally different from other people. | .40 | -0.05 | **0.64** | 0.02 | 0.00 |
| Item 7 | Someone with arthritis or a broken leg has just one thing wrong with them. but a person like Alex is fundamentally different from other people. | .48 | -0.07 | **0.75** | -0.01 | 0.05 |
| ~~Item 8~~ | Alex is in a state of mind that normal persons simply cannot understand. | .51 | 0.16 | **0.53** | -0.10 | -0.09 |
| Item 9 | People who have depression are fundamentally different from ordinary people. | .48 | -0.10 | **0.77** | -0.01 | 0.07 |
| Item 18 | People with mental health problems are normal people just like everyone else. | .58 | 0.00 | 0.26 | 0.02 | **0.53** |
| Item 12 | Depression is an "either/or" phenomenon - there is nothing in between | .28 | -0.02 | 0.11 | **0.36** | 0.08 |
| Item 19 | There is a fluid transition between mental health and mental illness. | .43 | 0.08 | 0.04 | **0.52** | -0.03 |
| Item 20 | Mental health and mental illness are separate and entirely different states. | .51 | -0.06 | 0.07 | **0.77** | -0.05 |
| Item 29 | There is a clear boundary between being mentally ill and being mentally healthy. | .52 | -0.03 | -0.19 | **0.78** | -0.05 |

*Note*. Item total r based on all the items considered for scale creation. Numbers according to the four emerged factors. Principal Axis Factoring with Promax rotation. Items marked with a strikethrough were excluded during the scale development process.

**S1.4 Final Items after study 1 (English / German):**

1. Most of us from time to time show symptoms of depression. /
   Ab und zu zeigen die meisten von uns Symptome einer Depression.
2. Given extreme circumstances, many of us could show signs of depression. /
   Unter extremen Umständen könnten viele von uns Anzeichen von Depression zeigen.
3. Everyone experiences mental health problems at some point; it’s just a matter of how severe these problems are. /
   Alle Menschen haben irgendwann einmal psychische Probleme, es ist nur die Frage, wie stark diese Probleme sind.
4. Someone with arthritis or a broken leg has just one thing wrong with them, but a person like Alex is fundamentally different from other people. /
   Bei einer Person mit Gelenkschmerzen oder einem Armbruch ist nur eine Sache nicht Ordnung, aber ein Mensch mit psychischen Problemen ist grundlegend verschieden von anderen Menschen.
5. People who have depression are fundamentally different from ordinary people. /
   Menschen mit Depression sind grundlegend anders als gewöhnliche Menschen.
6. People with mental health problems are normal people just like everyone else. /
   Menschen mit psychischen Problemen sind normale Menschen wie alle anderen auch.
7. There is a fluid transition between mental health and mental illness. /
   Zwischen psychischer Gesundheit und psychischer Krankheit gibt es einen fließenden Übergang.
8. Mental health and mental illness are separate and entirely different states. /
   Psychische Gesundheit und psychische Krankheit sind getrennte und vollkommen unterschiedliche Zustände.
9. There is a clear boundary between being mentally ill and being mentally healthy. /
   Es besteht eine klare Grenze zwischen psychischer Erkrankung und psychischer Gesundheit.
10. Basically we are all sometimes like this person, It’s just a question how pronounced this state is. /
    Im Grunde sind wir alle manchmal wie [Person X]. Es ist nur eine Frage, wie ausgeprägt dieser Zustand ist.
11. There is something about [Person X] that makes her fundamentally different from other people. /
    Es gibt etwas an [Person X], das sie grundlegend verschieden von anderen Menschen macht.
12. [Person X] can be either entirely mentally ill or mentally healthy – there is nothing in between. /
    [Person X] kann entweder vollständig psychisch krank oder psychisch gesund sein – es gibt nichts dazwischen.
    *(adapted item, not tested in study 1)*

**Supplement Phase 2**

**S 2.1 Cognitive Interview Guide Part Continuum Beliefs**

Interviewtext zum Vorlesen und Notieren
(INTERVIEWTEXT FOR READING ALOUD AND NOTATION)

**Instruktionen zum Leitfaden:**

- Du stellst immer dann Fragen, wenn sich der:die Proband:in bei dir meldet (es erscheint für Proband:in eine blaue Sprechblase und Hinweis, sich bei dir zu melden)

**In grauen Boxen siehst du, was der:die Proband:in gerade bearbeitet**

- *Text kursiv und unterstrichen:* Nur Hinweis, NICHT SAGEN
- „Text in Anführungszeichen: TEXT, DER GESAGT WERDEN SOLL (Muss nicht immer wortwörtlich sein!)“
- [[in eckigen Klammern stehen Fragen, die nach Bedarf und Gefühl gestellt werden können.]]
- Es ist generell ok und erwünscht, nach Gefühl nochmal nachzufragen oder Fragen zu wiederholen/umzuformulieren usw. ☺

***Instructions for the guide:***

- *Ask questions whenever the participant contacts you (a blue speech bubble will appear for the participant with a reminder to contact you)*
- *In gray boxes, you see what the participant is currently working on.*
- *Text in italics and underlined: Just a reminder, DO NOT SAY*
- *"Text in quotation marks: TEXT TO BE SAID (Doesn't always have to be verbatim!)"*
- *[[Questions in square brackets can be asked as needed and felt appropriate.]]*
- *It's generally okay and encouraged to ask again based on intuition or to repeat/rephrase questions, etc. ☺*

*Erklärungen zu Beginn des Telefoninterviews*

„Hallo, mein Name ist **[Name;** **kurz zu erzählen, wo man herkommt, was man arbeitet]** und ich werde heute mit Ihnen das Interview führen. Danke für Ihre Teilnahme an der Studie!

Sie können jetzt den Weblink öffne, den ich Ihnen in der Mail geschickt habe. Als Erstes bekommen Sie einen Einführungstext zu lesen und können Ihre Einwilligung abgeben.

*Explanations at the beginning of the telephone interview*

*Hello, my name is* ***[Name; briefly share where you're from, what you do professionally]****, and I will be conducting the interview with you today. Thank you for participating in the study! You can now open the web link that I sent you in the email. First, you will be presented with an introductory text to read and can provide your consent.*

**Continuum Beliefs**

**14.**

*Weglassen, wenn schon viel erzählt wurde*: „Was ging Ihnen bei den Fragen durch den Kopf? Sagen Sie bitte auch Dinge, die Ihnen vielleicht unwichtig erscheinen.“

*Omit if much has already been said: 'What went through your mind during the questions? Please also mention things that may seem unimportant to you.*

*Nachfragetechnik (Verständnis):* „Was verstehen Sie in dieser Frage unter dem Begriff ‚psychische Probleme‘?“

*Probe Technique (Understanding): "What do you understand by the term 'psychological problems' in this question?"*

[Wenn nötig: *Unspezifisches Nachfragen:* „Können Sie mir Ihre Antwort bitte noch etwas näher erläutern?“]

*[If necessary: Non-specific probing: "Could you please provide me with a more detailed explanation of your response?"]*

**Ende der Befragung (END OF INTERVIEW)**

„Wir sind jetzt am Ende der Befragung. Danke für Ihre Teilnahme!“

*Lautes Denken:* Wie ist Ihr genereller Eindruck der Studie?

*Lautes Denken:* Wie geht es Ihnen gerade?

*"We have now reached the end of the survey. Thank you for your participation!"*

*Thinking aloud: "What is your overall impression of the study?"
Thinking aloud: "How are you feeling right now?"*

„Vielen Dank für Ihre Teilnahme und Ihr Feedback. Wir möchten Ihnen gern als Dankeschön noch einen Gutschein in Höhe von 25 € (für den Online-Shop Grüne Bude) senden. Wir versenden die Gutscheine per E-Mai. Ist das für Sie in Ordnung? Ihre E-Mail-Adresse habe ich ja schon, oder?“

[*Eventuell E-Mail-Adresse notieren.*]

*"Thank you very much for your participation and feedback. We would like to send you a voucher worth 25€ (for the online shop 'Grüne Bude') as a token of appreciation. We will send the vouchers via email. Is that okay for you? I already have your email address, right?"*

*[Possibly note down the email address.]*

„Ich wünsche Ihnen noch einen angenehmen Tag. Tschüss ☺“

*I wish you a pleasant day. Goodbye* ☺

**S2.2 Case Vignette & Intervention**

***Case Vignette***

Hello,

I’m NAME and I’d like to tell you about my recent experiences. For a few months now I’ve often felt sad and can’t even explain what’s been pulling me down. It’s been really frustrating!

I rarely experience joy anymore and spending time with my friends isn’t as fun as it used to be. On some days I already struggle with minor decisions in the morning, such as choosing what to wear or what I’ll have for breakfast.

I find it hard to pull myself together to leave the house and I have trouble concentrating on my work. I often find myself worrying extensively about little things and I blame myself for everything and anything. I sometimes struggle with myself, even though I have come to realise that these are signs of depression.

***Intervention: Introduction***

You’ve already gotten to know NAME a little bit and might be able to understand her/him/them.

NAME has followed up on his/her/their depression. Some realisations have given him/her/them courage and helped her/him/them so much that he/she/them would like to share these with you.

On the following pages you will gain insight into NAME’s personal experiences and thoughts.

You will repeatedly have the opportunity to reflect on what you might be able to take away from NAME’s experiences. We would like to encourage you to think about which of NAMES’ experiences might be particularly relevant for you personally.

***Intermediary Variables – All Texts & Questions to prompt reflection***

Continuum Beliefs

I wondered: Am I different now? Am I not normal anymore? Of course, everyone experiences negative feelings and internal stress from time to time, especially in difficult situations. The question, rather, is how long those negative feelings last for and how much of a burden they are. Sometimes, when I feel particularly bad I forget that I’ve ever felt better and I can’t imagine feeling good in the future.

I find it helpful to think of a scale all people are located on. Nobody is 100% healthy or sick, everyone is situated somewhere in between. There isn’t an all or nothing, no “normal” or “abnormal”, but rather fluid transitions. A depression can change and is treatable.

However, to support people with these changes, it’s important to have descriptions for this condition. To label the condition as “depression” helps to identify and treat it.

*…Continuum Beliefs – question*

Take a moment to think about what you just read/saw.

How would you describe your mental health right now? Please move the slider to the fitting position.

1 2 3 4 5 6 7

(I’m currently feeling sick) (I’m currently feeling healthy)

|  | **Table S2.2**  *Continuum Beliefs of Mental Illness Scale: Items ordered to the Subscales (Vignette-Related Items as Additional Subgroup)*  *(Introduction)* The following questions concern your opinion on mental illnesses. Please indicate the extent to which you agree or disagree with each statement. 1 (strongly disagree) to 5 (strongly agree) | | | | | | | | | | |
| --- | --- | --- | --- | --- | --- | --- | --- | --- | --- | --- | --- |
|  | Items | | | | | | Formulation | | | | |
| **Subscale State** | | | | |  | | | | | |  |
| 1 | | Most of us from time to time show symptoms of mental illness. | | | |  | | Inside Perspective, Similarity | | | |
| 2 | | Given extreme circumstances, many of us could show signs of mental health problems. | | | |  | | Inside Perspective, Similarity | | | |
| 3 | | Everyone experiences mental health problems at some point; it's just a matter of how severe these problems are. | | | |  | | Inside Perspective, Similarity | | | |
|  | |  | | | |  | |  | | | |
| **Subscale Person** | | | |  | | | | | |  |  |
| 4 | | Someone with arthritis or a broken leg has just one thing wrong with them,  but a person with mental illness is fundamentally different from other people. | | | |  | | Outside Perspective, Differentness | | | |
| 5 | | People who have a mental illness are fundamentally different from ordinary people. | | | |  | | Outside Perspective, Differentness | | | |
| 6 | | People with mental health problems are normal people just like everyone else. | | | |  | | Outside Perspective, Similarity | | | |
|  | |  | | | |  | |  | | | |
| **Subscale Concept** | | | |  | | | | | |  |  |
| 7 | | There is a fluid transition between mental health and mental illness. | | | |  | | Outside Perspective, Similarity | | | |
| 8 | | Mental health and mental illness are separate and entirely different states. | | | |  | | Outside Perspective, Differentness | | | |
| 9 | | There is a clear boundary between being mentally ill and being mentally healthy. | | | |  | | Outside Perspective, Differentness | | | |
|  | |  | | | |  | |  | | | |
| **Optional Subscale Vignette-related (Post-Intervention Test)** | | |  | | | | | |  |  |  |
| 10 | | Basically we are all sometimes like [person X]. It’s just a question how pronounced this state is. | | | |  | | Outside Perspective, Similarity | | | |
| 11 | | There is something about [person X] that makes them fundamentally different from other people. | | | |  | | Outside Perspective, Differentness | | | |
| 12 | | [Person X] can be either entirely mentally ill or mentally healthy – there is nothing in between. | | | |  | | Outside Perspective, Differentness | | | |
| *German Original Version* | | | | | | | | | | | |
|  | | | | | | | | | | | |
| (*Instruktion* ) In den folgenden Fragen geht es um Ihre Meinung zu psychischen Krankheiten. Bitte geben Sie an, inwieweit Sie der jeweiligen Aussage zustimmen oder nicht. | | | | | | | | | | | |

| **Subskala Symptomorientierung** | | | | | |
| --- | --- | --- | --- | --- | --- |
| 1 | Ab und zu zeigen die meisten von uns Symptome einer psychischen Erkrankung. | | | |  |
| 2 | Unter extremen Umständen könnten viele von uns Anzeichen von psychischen Problemen zeigen. | | | |  |
| 3 | Alle Menschen haben irgendwann einmal psychische Probleme, es ist nur die Frage wie stark diese Probleme sind. | | | |  |
| **Subskala Normorientierung** | | | |  |  |
| 4 | Bei einer Person mit Gelenkschmerzen oder einem Armbruch ist nur eine Sache nicht in Ordnung,  aber ein Mensch mit psychischen Problemen ist grundlegend verschieden von anderen Menschen. | | | |  |
| 5 | Menschen mit psychischen Problemen sind grundlegend anders als gewöhnliche Menschen. | | | |  |
| 6 | Menschen mit psychischen Problemen sind normale Menschen wie alle anderen auch. | | | |  |
| **Subskala Kontinuumskonzept** | | |  |  |  |
| 7 | Zwischen psychischer Gesundheit und psychischer Krankheit gibt es einen fließenden Übergang. | | | |  |
| 8 | Psychische Gesundheit und psychische Krankheit sind getrennte und vollkommen unterschiedliche Zustände. | | | |  |
| 9 | Es gibt eine klare Grenze zwischen psychischer Krankheit und psychischer Gesundheit. | | | |  |
| **Optionale Subskala Vignettenbezug (Posttestung)** | |  |  |  |  |
| 10 | Im Grunde sind wir alle manchmal wie [Person X]. Es ist nur eine Frage, wie ausgeprägt dieser Zustand ist. | | | |  |
| 11 | Es gibt etwas an [Person X], das [sie/ihn] grundlegend verschieden von anderen Menschen macht. | | | |  |
| 12 | [Person X] kann entweder komplett psychisch krank oder psychisch gesund sein - es gibt nichts dazwischen. | | | |  |

*Note*. New Numbers assigned to the items of this finals scale. State= Normality of being in a state of having mental health problems, Person=Normality of individuals with mental health problems. Concept=Nosological conecpt of a continuum of mental health and illness. Items 10, 11, 12 each assigned to the facets of subscales State, Person, Concept. Content Grouping from Table S1.1 in terms of In-Group vs. Out-group, and Similarity vs. Differentness.

**Table S2.3**

*Descriptives item statistics of the Continuum Beliefs of Mental Illness Scale – Timepoint 1 (N=1375)*

|  | Items | *M* | *SD* | Skewness | Kurtosis | Item total *r* |
| --- | --- | --- | --- | --- | --- | --- |
| 1 | Most of us from time to time show symptoms of mental illness. | 3.76 | 0.92 | -0.46 | -0.10 | 0.58 |
| 2 | Given extreme circumstances, many of us could show signs of mental health problems. | 4.21 | 0.82 | -0.92 | 0.66 | 0.51 |
| 3 | Everyone experiences mental health problems at some point; it's just a matter of how severe these problems are. | 4.03 | 0.97 | -0.91 | 0.41 | 0.53 |
| 4 | Someone with arthritis or a broken leg has just one thing wrong with them, but a person with mental illness is fundamentally different from other people. | 3.02 | 1.29 | 0.08 | -1.04 | 0.41 |
| 5 | People who have a mental illness are fundamentally different from ordinary people. | 3.70 | 1.20 | -0.58 | -0.63 | 0.56 |
| 6 | People with mental health problems are normal people just like everyone else. | 4.21 | 0.99 | -1.19 | 0.85 | 0.39 |
| 7 | There is a fluid transition between mental health and mental illness. | 3.66 | 1.01 | -0.42 | -0.17 | 0.32 |
| 8 | Mental health and mental illness are separate and entirely different states. | 3.42 | 1.17 | -0.27 | -0.66 | 0.43 |
| 9 | There is a clear boundary between being mentally ill and being mentally healthy. | 3.43 | 1.15 | -0.34 | -0.53 | 0.48 |

*Note*. *M* = mean score. *SD*=standard deviation*. r* = item total correlation with the subscale the respective item is assigned to.

**Table S2.4**

*Correlation Coefficients of 9 Items of the Continuum Beliefs of Mental Illness Scale – Timepoint 1 (N=1375)*

| Items | 1 | 2 | 3 | 4 | 5 | 6 | 7 | 8 |
| --- | --- | --- | --- | --- | --- | --- | --- | --- |
| 2 | .48** |  |  |  |  |  |  |  |
| 3 | .49** | .41** |  |  |  |  |  |  |
| 4 | .04 | .05* | -.02 |  |  |  |  |  |
| 5 | .05 | .10** | .04 | .44** |  |  |  |  |
| 6 | .20** | .28** | .20** | .23** | .44** |  |  |  |
| 7 | .27** | .31** | .23** | .01 | .07** | .22** |  |  |
| 8 | .08** | .11** | .03 | .22** | .26** | .15** | .24** |  |
| 9 | .01 | .08** | .01 | .26** | .27** | .08** | .30** | .44** |

*Note.* Spearman correlation coefficients. * indicates *p* < .05. ** indicates *p* < .01.

**Table S2.5**

*Correlation Coefficients of 12 Items of the Continuum Beliefs of Mental Illness Scale – Timepoint 2(N=1375)*

| Item | 1 | 2 | 3 | 4 | 5 | 6 | 7 | 8 | 9 | 10 | 11 | 12 |
| --- | --- | --- | --- | --- | --- | --- | --- | --- | --- | --- | --- | --- |
| 1 |  |  |  |  |  |  |  |  |  |  |  |  |
| 2 | **.54**** |  |  |  |  |  |  |  |  |  |  |  |
| 3 | **.52**** | **.47**** |  |  |  |  |  |  |  |  |  |  |
| 4 | .01 | .07* | -.01 |  |  |  |  |  |  |  |  |  |
| 5 | .09** | .18** | .09** | **.48**** |  |  |  |  |  |  |  |  |
| 6 | .21** | **.33**** | .21** | .18** | **.38**** |  |  |  |  |  |  |  |
| 7 | .28** | **.32**** | .25** | .07* | .11** | .21** |  |  |  |  |  |  |
| 8 | .14** | .13** | .09** | **.31**** | **.32**** | .14** | **.34**** |  |  |  |  |  |
| 9 | .14** | .14** | .06* | **.31**** | **.35**** | .11** | **.35**** | **.54**** |  |  |  |  |
| 10 | **.38**** | **.39**** | **.49**** | .03 | .14** | .17** | .20** | .11** | .07** |  |  |  |
| 11 | .13** | .22** | .10** | **.42**** | **.51**** | .27** | .16** | .29** | **.31**** | .15** |  |  |
| 12 | .14** | .21** | .09** | .28** | .**39**** | .25** | .21** | **.35**** | **.40**** | .10** | **.43**** |  |

*Note.* In bold: Spearman correlation coefficients ≥.30. * indicates *p* < .05. ** indicates *p* < .01.

**Table 2.6**

*Loadings of Exploratory and Confirmatory Factor Analyses of the Nine-Item and 12-Item Version of the Continuum Beliefs of Mental Illness-Scale, Timepoint 1 and 2 (N=1375)*

|  | 9-Item Version (Timepoint 1) | | | | 12-Item version (Timepoint 2) | | |
| --- | --- | --- | --- | --- | --- | --- | --- |
|  | EFA | | | CFA | EFA | | |
| Item | State | Person | Concept |  | State | Person | Concept |
| 1 | **0.76** | -0.03 | -0.04 | .750 | **0.70** | -0.12 | 0.13 |
| 2 | **0.69** | 0.03 | 0.02 | .688 | **0.69** | 0.08 | 0.06 |
| 3 | **0.70** | -0.02 | -0.09 | .648 | **0.72** | -0.01 | -0.07 |
| 4 | -0.09 | **0.48** | 0.13 | .556 | -0.19 | **0.53** | 0.13 |
| 5 | -0.09 | **0.83** | 0.02 | .931 | -0.04 | **0.90** | -0.07 |
| 6 | 0.28 | **0.52** | -0.12 | .702 | 0.27 | **0.51** | -0.11 |
| 7 | 0.35 | -0.06 | **0.33** | .690 | 0.29 | -0.15 | **0.55** |
| 8 | -0.02 | 0.14 | **0.51** | .706 | -0.08 | 0.08 | **0.68** |
| 9 | -0.09 | -0.04 | **0.86** | .824 | -0.10 | 0.09 | **0.72** |
| 10 |  |  |  |  | **0.57** | 0.07 | -0.08 |
| 11 |  |  |  |  | 0.08 | **0.60** | 0.04 |
| 12 |  |  |  |  | 0.03 | 0.36 | **0.29** |

*Note*. EFA=Exploratory Factor Analysis: 9-Item Version: KMO (Kaiser-Meyer-Olkin measure) =.71; correlations of subscales: State – Person: *r*=.24; Person-Concept: *r*=.41; State-Concept: *r*=.20. CFA=Confirmatory Factor Analysis: Displayed Loading refer to the factor, the items are assigned to. CFI = .970; TLI= .943; RMSEA= .048; SRMR= .039; Loading of subscales on higher order factor: State = .701; Person = .715; Concept = .769. 12-item version: EFA: KMO = .81, correlations of subscales: State – Person: *r*=.25; Person-Concept: *r*=.51; State-Concept: *r*=.26. in Bold: Loadings on the respective factor, the items are assigned to.

**Table S2.7**

*Correlation Coefficients of Continuum Beliefs of Mental Illness Scale Single Items with Stigma and Health-Related Variables at Timepoint 1 (N=1375)*

|  | Depression severity | Self-identi-fication | Help-Seeking Attitudes | Stereotype awareness | Stereotype agreement | Agree-ment to  Discrimi-nation | Shame | Social distance | Blame | Fear | Anger | Pro-social reactions |
| --- | --- | --- | --- | --- | --- | --- | --- | --- | --- | --- | --- | --- |
| 1 | .030 | .114^**^ | .087^**^ | .059^*^ | -.077^**^ | -.132^**^ | -.082^**^ | -.134^**^ | -.105^**^ | -.102^**^ | -.058^*^ | .078^**^ |
| 2 | .004 | .127^**^ | .178^**^ | .031 | -.160^**^ | -.147^**^ | -.123^**^ | -.189^**^ | -.201^**^ | -.110^**^ | -.141^**^ | .106^**^ |
| 3 | -.003 | .010 | .079^**^ | .021 | -.065^*^ | -0.040 | -.097^**^ | -.076^**^ | -.078^**^ | -.042 | -.048 | .079^**^ |
| 4 | -.081^**^ | -.059^*^ | .053 | -.010 | -.133^**^ | -.153^**^ | -.028 | -.133^**^ | -.134^**^ | -.079^**^ | -.079^**^ | -.050 |
| 5 | -.096^**^ | .000 | .122^**^ | -.010 | -.280^**^ | -.229^**^ | -.118^**^ | -.234^**^ | -.287^**^ | -.227^**^ | -.221^**^ | .033 |
| 6 | -.006 | .147^**^ | .188^**^ | -.035 | -.358^**^ | -.318^**^ | -.172^**^ | -.351^**^ | -.357^**^ | -.280^**^ | -.284^**^ | .122^**^ |
| 7 | .001 | .131^**^ | .148^**^ | .012 | -.129^**^ | -.126^**^ | -.072^**^ | -.144^**^ | -.156^**^ | -.046 | -.091^**^ | .084^**^ |
| 8 | -.059^*^ | .102^**^ | .170^**^ | .007 | -.148^**^ | -.147^**^ | -.089^**^ | -.103^**^ | -.173^**^ | -.101^**^ | -.096^**^ | .021 |
| 9 | -.036 | .116^**^ | .077^**^ | .060^*^ | -.139^**^ | -.138^**^ | -.023 | -.108^**^ | -.193^**^ | -.056^*^ | -.093^**^ | .009 |

*Note.* Spearman correlation coefficients.1-9 = Single Items. * indicates *p* < .05. ** indicates *p* < .01.

**Table S2.8**

*Correlation Coefficients of Continuum Beliefs of Mental Illness Single Items with Stigma and Health-Related Variables at Timepoint 2 Post-intervention (N=1375)*

|  | Depression severity | Self-identi-fication | Help-Seeking Attitudes | Stereotype awareness | Stereotype agreement | Agree-ment to  Discrimination | Shame | Social distance | Blame | Fear | Anger | Pro-social reactions |
| --- | --- | --- | --- | --- | --- | --- | --- | --- | --- | --- | --- | --- |
| 1 | .077^**^ | .192^**^ | .167^**^ | .102^**^ | -.092^**^ | -.123^**^ | -.135^**^ | -.160^**^ | -.140^**^ | -.118^**^ | -.105^**^ | .158^**^ |
| 2 | .056^*^ | .178^**^ | .218^**^ | .120^**^ | -.195^**^ | -.175^**^ | -.163^**^ | -.208^**^ | -.213^**^ | -.172^**^ | -.172^**^ | .181^**^ |
| 3 | .041 | .081^**^ | .173^**^ | .095^**^ | -.099^**^ | -.075^**^ | -.133^**^ | -.150^**^ | -.099^**^ | -.096^**^ | -.096^**^ | .151^**^ |
| 4 | -.037 | .012 | .110^**^ | -.003 | -.247^**^ | -.206^**^ | -.077^**^ | -.209^**^ | -.196^**^ | -.152^**^ | -.130^**^ | .001 |
| 5 | -.027 | .137^**^ | .236^**^ | -.044 | -.483^**^ | -.377^**^ | -.168^**^ | -.371^**^ | -.390^**^ | -.334^**^ | -.325^**^ | .098^**^ |
| 6 | .003 | .148^**^ | .239^**^ | -.024 | -.391^**^ | -.337^**^ | -.240^**^ | -.440^**^ | -.311^**^ | -.364^**^ | -.354^**^ | .139^**^ |
| 7 | -.003 | .141^**^ | .184^**^ | .057^*^ | -.175^**^ | -.147^**^ | -.092^**^ | -.194^**^ | -.198^**^ | -.109^**^ | -.157^**^ | .105^**^ |
| 8 | -.062^*^ | .122^**^ | .203^**^ | .033 | -.231^**^ | -.206^**^ | -.094^**^ | -.173^**^ | -.222^**^ | -.136^**^ | -.144^**^ | .023 |
| 9 | -.021 | .140^**^ | .192^**^ | .057^*^ | -.240^**^ | -.229^**^ | -.068^*^ | -.186^**^ | -.251^**^ | -.148^**^ | -.165^**^ | .021 |
| 10 | -.019 | .072^**^ | .126^**^ | .081^**^ | -.124^**^ | -.104^**^ | -.072^**^ | -.115^**^ | -.095^**^ | -.092^**^ | -.124^**^ | .164^**^ |
| 11 | -.060^*^ | .091^**^ | .152^**^ | -.006 | -.340^**^ | -.257^**^ | -.144^**^ | -.241^**^ | -.301^**^ | -.261^**^ | -.262^**^ | .047 |
| 12 | -.052 | .164^**^ | .205^**^ | .003 | -.331^**^ | -.307^**^ | -.140^**^ | -.262^**^ | -.346^**^ | -.283^**^ | -.288^**^ | .092^**^ |

*Note.* Spearman correlation coefficients . CB = mean score of continuum beliefs scale.1-12 = Single Items. * indicates *p* < .05. ** indicates *p* < .01

**Table S2.9**

|  |  | Overall scale | State | Person | Concept |
| --- | --- | --- | --- | --- | --- |
| Stereotype awareness | 12 Item-version | .069^*^ | .128^**^ | -.019 | .050 |
|  | 9 Item-version | .016 | .057^*^ | -.025 | .043 |
| Stereotype agreement | 12 Item-version | **-.398^**^** | -.154^**^ | **-.462^**^** | **-.319^*^** |
|  | 9 Item-version | -.298^**^ | -.112^**^ | -.325^**^ | -.182^**^**^*^** |
| Discrimination | 12 Item-version | **-.354^**^** | -.144^**^ | **-.380^**^** | -.291^**^ |
|  | 9 Item-version | -.292^**^ | -.126^**^ | -.297^**^ | -.178^**^ |
| Shame | 12 Item-version | -.206^**^ | -.161^**^ | -.193^**^ | -.125^**^ |
|  | 9 Item-version | -.155^**^ | -.118^**^ | -.132^**^ | -.078^**^ |
| Social distance | 12 Item-version | **-.384^**^** | -.194^**^ | -.394^**^ | -.265^**^ |
|  | 9 Item-version | -.289^**^ | -.157^**^ | -.298^**^ | -.150^**^ |
| Blame | 12 Item-version | **-.376^**^** | -.173^**^ | **-.386^**^** | **-.327^**^** |
|  | 9 Item-version | -.341^**^ | -.149^**^ | -.328^**^ | -.229^**^ |
| Fear | 12 Item-version | -.288^**^ | -.150^**^ | **-.346^**^** | -.218^**^ |
|  | 9 Item-version | -.201^**^ | -.102^**^ | -.243^**^ | -.088^**^ |
| Anger | 12 Item-version | -.299^**^ | -.152^**^ | **-.337^**^** | -.249^**^ |
|  | 9 Item-version | -.219^**^ | -.095^**^ | -.242^**^ | -.120^**^ |
| Pro-social reactions | 12 Item-version | .142^**^ | .199^**^ | .082^**^ | .077^**^ |
|  | 9 Item-version | .082^**^ | .108^**^ | .036 | .039 |
| Depression severity | 12 Item-version | -.012 | .040 | -.050 | -.055^*^ |
|  | 9 Item-version | -.073^**^ | .007 | -.086^**^ | -.045 |
| Self-identification | 12 Item-version | .211^**^ | .160^**^ | .118^**^ | .178^**^ |
|  | 9 Item-version | .130^**^ | .102^**^ | .025 | .153^**^ |
| HS Attitudes | 12 Item-version | **.320^**^** | .214^**^ | .230^**^ | .257^**^ |
|  | 9 Item-version | .223^**^ | .143^**^ | .153^**^ | .171^**^ |

*Correlation Coefficients of Continuum Beliefs of Mental Illness Global Scores and Subscale Scores of the 9-Item (Timepoint 1) and 12-Item (Timepoint 2 Post-intervention) versions with Stigma and Health-Related Variables.
Note*. Spearman correlation coefficients. HS = Help-Seeking. * indicates *p* < .05. ** indicates *p* < .01.
